# Lipidomic Response to Coffee Consumption

**DOI:** 10.3390/nu10121851

**Published:** 2018-12-01

**Authors:** Alan Kuang, Iris Erlund, Christian Herder, Johan A. Westerhuis, Jaakko Tuomilehto, Marilyn C. Cornelis

**Affiliations:** 1 Department of Preventive Medicine, Northwestern University Feinberg School of Medicine, 680 North Lake Shore Drive, Suite 1400, Chicago, IL 60611, USA; alan.kuang@northwestern.edu; 2 Genomics and Biomarkers Unit, National Institute for Health and Welfare, P.O. Box 30, 00271 Helsinki, Finland; iris.erlund@thl.fi; 3 Institute for Clinical Diabetology, German Diabetes Center, Leibniz Center for Diabetes Research at Heinrich Heine University Düsseldorf, 40225 Düsseldorf, Germany; Christian.Herder@DDZ.UNI-DUESSELDORF.DE; 4 German Center for Diabetes Research (DZD), 85764 München-Neuherberg, Germany; 5 Biosystems Data Analysis, Swammerdam Institute for Life Sciences, University of Amsterdam, Science Park 904, 1098 XH Amsterdam, The Netherlands; j.a.westerhuis@uva.nl; 6 Centre for Human Metabolomics, Faculty of Natural Sciences, North-West University (Potchefstroom Campus), Private Bag X6001, Potchefstroom 2520, South Africa; 7 Disease Risk Unit, National Institute for Health and Welfare, 00271 Helsinki, Finland; jaakko.tuomilehto@thl.fi; 8 Department of Public Health, University of Helsinki, 00014 Helsinki, Finland; 9 Saudi Diabetes Research Group, King Abdulaziz University, 21589 Jeddah, Saudi Arabia

**Keywords:** coffee, caffeine, lipids, biomarkers, trial, lysophosphatidylcholine, lipidomics

## Abstract

Coffee is widely consumed and contains many bioactive compounds, any of which may impact pathways related to disease development. Our objective was to identify individual lipid changes in response to coffee drinking. We profiled the lipidome of fasting serum samples collected from a previously reported single blinded, three-stage clinical trial. Forty-seven habitual coffee consumers refrained from drinking coffee for 1 month, consumed 4 cups of coffee/day in the second month and 8 cups/day in the third month. Samples collected after each coffee stage were subject to quantitative lipidomic profiling using ion-mobility spectrometry–mass spectrometry. A total of 853 lipid species mapping to 14 lipid classes were included for univariate analysis. Three lysophosphatidylcholine (LPC) species including LPC (20:4), LPC (22:1) and LPC (22:2), significantly decreased after coffee intake (*p* < 0.05 and *q* < 0.05). An additional 72 species mapping to the LPC, free fatty acid, phosphatidylcholine, cholesteryl ester and triacylglycerol classes of lipids were nominally associated with coffee intake (*p* < 0.05 and *q* > 0.05); 58 of these decreased after coffee intake. In conclusion, coffee intake leads to lower levels of specific LPC species with potential impacts on glycerophospholipid metabolism more generally.

## 1. Introduction

Coffee is one of the most widely consumed beverages in the world and has been implicated in numerous diseases such as type 2 diabetes (T2D) and cardiovascular disease [1,2,3,4]. The causal and precise molecular mechanisms that underlie the beneficial and adverse effects of coffee remain unclear. Coffee is the major source of caffeine for many populations [5], but it also contains hundreds of other compounds, many of which might impact pathways related to disease development or prevention [6].

High-throughput omic profiling techniques enable thorough studies of an individual’s response to coffee intake and provide potentially new mechanistic insight to the role coffee plays in health [7,8]. We recently performed a comprehensive metabolomics study of coffee consumption leveraging serum samples collected during a coffee trial [8,9]. Over 100 metabolites were significantly associated with coffee intake; several mapping to xanthine, benzoate, steroid, endocannabinoid and fatty acid (acylcholine) metabolism. We extend this work to comprehensive lipid profiling for the first time. Lipid molecules are a subset of the metabolome and serve as ubiquitous and essential multifunctional metabolites [10]. Lipids are directly exposed to intracellular and extracellular biochemical changes and as a result undergo various modifications themselves [10]. Our objective was to identify individual lipid changes in response to coffee in order to gain more insight into biological mechanisms by which coffee may impact health.

## 2. Subjects and Methods

### 2.1. Coffee Trial

Serum samples analyzed in the current study were obtained from participants completing an investigator-blinded, three-stage controlled trial in 2009–2010 that lasted for 3 months (Supplementary Note 1, ISRCTN registry: ISRCTN12547806) [9]. Briefly, habitual coffee consumers <65 years of age, residing in Finland, free of T2D, but with an elevated risk of T2D were eligible for participation. The participants received packages of coffee and brewed the coffee daily at home with their own coffee machine using paper filters. During the first month, participants refrained from drinking coffee, whereas in the second month they were instructed to consume 4 cups coffee/day (1 cup = 150 mL, Juhla Mokka brand) and in the third month 8 cups/day. Of the 49 participants recruited, 47 completed the trial. Baseline characteristics of these 47 participants are shown in Table S1. Several clinical biomarkers were measured and analyzed as part of the initial report as previously described [9]. Serum concentrations of total cholesterol, High-density lipoprotein (HDL) cholesterol, apo A-I and adiponectin increased significantly in response to coffee intake, whereas interleukin-18, 8-isoprostane, and the ratios of low-density lipoprotein (LDL) to HDL cholesterol and of apo B and apo A-I decreased significantly. The trial was conducted in accordance with the Declaration of Helsinki (1964), as amended in South Africa (1996), and approved by Joint Authority for the Hospital District of Helsinki and Uusimaa Ethics Committee, Department of Medicine, Helsinki, Finland. Written informed consent was obtained from all participants.

### 2.2. Lipidomics Assay, Data Acquisition and Processing

Lipid species were measured in fasting serum samples collected after each coffee stage (True Mass Complex Lipid Panel, Metabolon, Research Triangle Park, NC, USA). Lipids were extracted from samples using dichloromethane and methanol in a modified Bligh-Dyer extraction in the presence of internal standards with the lower, organic, phase being used for analysis. The extracts were concentrated under nitrogen and reconstituted in 0.25 mL of dichloromethane:methanol (50:50) containing 10 mM ammonium acetate. The extracts were placed in vials for infusion-mass spectrometry (MS) analyses, performed on a SelexION equipped Sciex 5500 QTRAP using both positive and negative mode electrospray. Each sample was subjected to two analyses, with ion mobility spectrometry (IMS)-MS conditions optimized for lipid classes monitored in each analysis. The 5500 QTRAP was operated in MRM mode to monitor the transitions for over 1100 lipids from up to 14 lipid classes including cholesteryl esters (CE), triacylglycerols (TAG), diacylglycerols (DAG), free fatty acids (FFA), phosphatidylcholines (PC), phosphatidylethanolamines (PE), phosphatidylinositols (PI), lysophosphatidylcholines (LPC), lysophosphatidylethanolamines (LPE), sphingomyelin (SM), ceramide (CER), hexosylceramides (HCER), lactosylceramides (LCER), dihydroceramides (DCER). Individual lipid species were quantified based on the ratio of signal intensity for target compounds to the signal intensity for an assigned internal standard of known concentration. Missing values were imputed with the observed minimum value. Individual lipid species that contained more than 20% missing values across the first (0 cups/day) and third (8 cups/day) trial stages were not included for statistical analysis (120 lipid species, Table S2) leaving a total of 853 lipid species for analysis. The same data, but with missing values treated as 0, were also expressed as mole% determined by calculating the proportion of individual species within each class. In secondary analysis, lipid species data were used to derive additional and biologically meaningful lipid traits. Lipid class concentrations were calculated from the sum of all molecular species within a class. For lipid classes containing more than one fatty acid (FA) per species (i.e., DAG, PC, PE, PI, and TAG) we also determined FA concentrations by calculating the sum of individual FAs within each of these classes. These traits were derived prior to excluding the lipids in Table S2 (see above). The final set of lipid species (primary traits) and derived lipid traits (secondary) analyzed in the current study are listed in Table S3.

### 2.3. Statistical Analysis

All statistical analyses were performed using R, SAS version 9.4 (SAS Institute Inc, Cary, NC, USA) or Matlab. To explore the data and identify any outlier samples we first performed standard principal component analysis (PCA) and multilevel PCA [11]. For the latter, we generated a data matrix of the within-person variation by subtracting individual lipid values from the mean lipid value of all three coffee stages, per participant, per lipid. Repeated measures ANOVA was used to test the relationship between coffee treatment and each individual lipid species. P-values were further adjusted for multiple comparisons by the Benjamini–Hochberg procedure and the false discovery rate (FDR)-adjusted P-values, expressed as *q*-values, are reported [12]. All nominal (*p* < 0.05) associations are presented but only those with a *q*-value < 0.05 are defined as statistically significant. We computed ordinary Pearson correlations to explore the latent relationships of changes in identified coffee lipids across treatments. These analyses were additionally supplemented with data for metabolites and clinical biomarkers that previously changed in response to coffee in this coffee trial (Table S4) [8,9]. Formal cross-platform integration analysis will be a focus of another report. Correlation networks were constructed using Cytoscape [13]. In secondary analysis, lipid class and fatty acid concentrations were also subject to univariate analysis. A multivariate approach was also pursued as traditionally done with high-throughput data and is presented in Supplementary Note 2 and Figure S5.

## 3. Results

PCA or multilevel-PCA demonstrated no clear separation of samples by coffee stage (Figure S1). As a result, no clear outliers were detected and thus all samples were included for our primary analysis.

**Figure 1:**
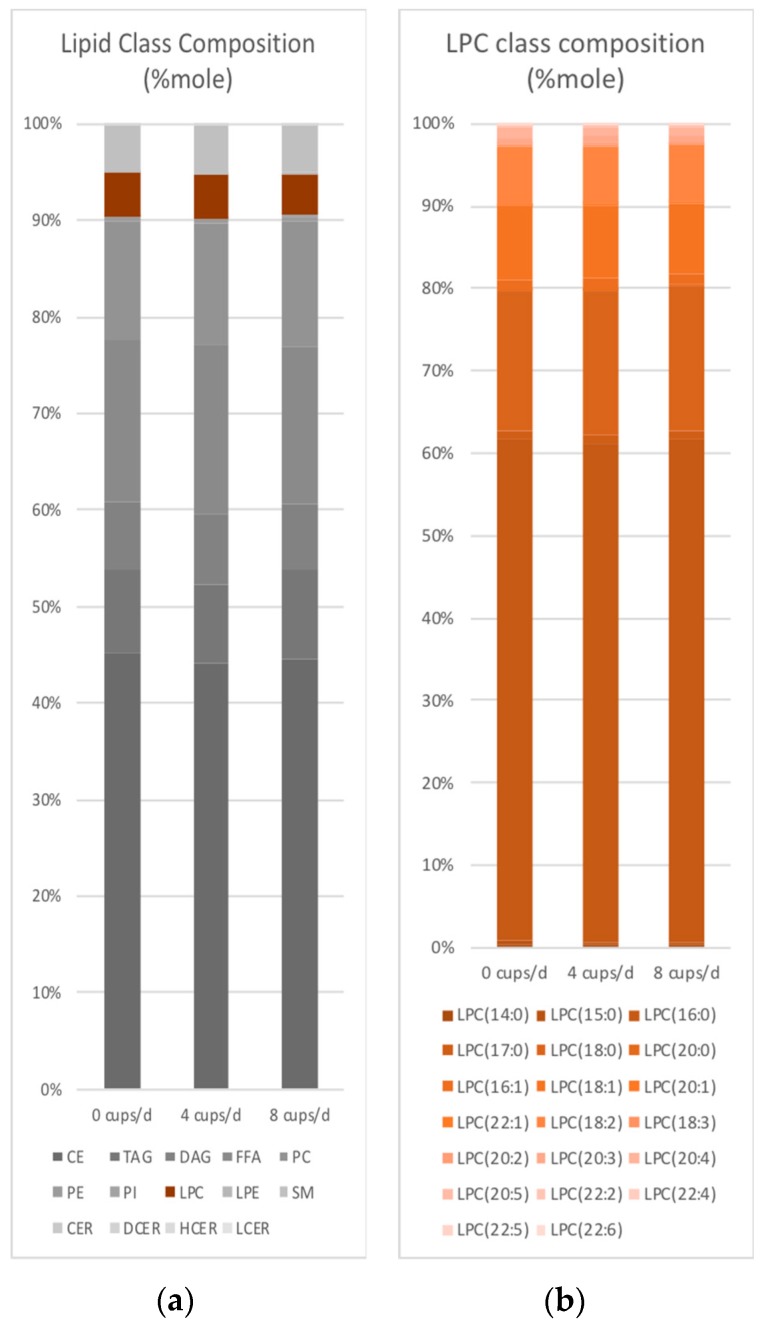
Lipid class (**a**) and LPC (**b**) composition response to coffee intake.

**Figure 2:**
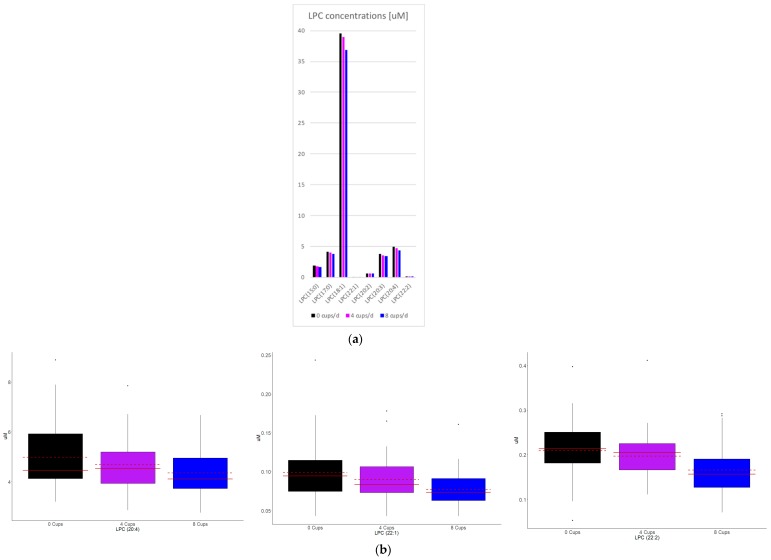
LPC concentration response to coffee intake. Shown are all nominally (**a**) to significant (**a**,**b**) LPCs that changed in response to coffee.

Serum lipid class concentrations (data not shown) or distributions (Figure 1a) did not significantly change in response to coffee intake. A total of 75 lipid species were at least nominally associated with coffee intake and these mapped to 8 lipid classes (*p* < 0.05, Table 1, Figure 2a and Figure S2). When applying an FDR correction, LPC 20:4, 22:1 and 22:2 remained significantly associated with coffee intake (Figure 2b). Similar results were observed when lipid species concentrations were expressed as mole% (Figure 1b and data not shown). When FA concentrations of DAG, PC, PE, PI, and TAG were examined, no associations met statistical significance (data not shown).

Results of correlation analysis of changes among previously identified clinical [9] and metabolite [8] markers of coffee response and the 75 nominal to significant lipid species identified here (Table 1) are presented in Figure S3. Generally lipid species of the same class or sharing fatty acid chains clustered together. Changes in TAGs that increased in response to coffee, however, did not correlate with changes in TAGs that decreased in response to coffee. Changes in lipid species generally correlated with metabolites that also decreased in response to coffee and thus unlikely originated from the coffee beverage itself. These metabolites were also lipid derivatives; particularly those of the acyl choline and endocannabinoid pathways. Besides kynurenine and xanthines, few other aqueous metabolites were consistently represented among correlations with either clinical makers or lipid species. No changes in lipids or metabolites were consistently correlated with clinical markers that responded to coffee.

## 4. Discussion

The current study is the first controlled trial-based lipidomic assessment of coffee intake. We found three LPC species (LPC (20:4), LPC (22:1) and LPC (22:2)) that significantly decreased after 4 and 8 cups per day. Several other species mapping to the LPC, FFA, PC and CE classes showed nominal but plausible changes. Although the current lipid species analysis is unique from that of our previous metabolomic analysis [8] of the same samples the findings taken together suggest that coffee drinking has more of an immediate impact on non-lipid than lipid metabolites over the duration of the coffee trial examined here.

The lipidomics platform was unable to distinguish between fatty acid isoforms, their position on a glycerol backbone (i.e., sn-1 vs. sn-2) or define their bond type (acyl- or alkyl-). Several lipid species at least nominally associated with coffee response contained FA(20:4). In our previous metabolomics report [8], arachidonic acid (AA, 20:4n6) and LPC (20:4n6) were specifically measured and decreased in response to coffee (*p* < 0.05, *q* < 0.05 for AA and *p* < 0.05, *q* > 0.05 for LPC (20:4n6)). These findings, along with the correlation patterns among these lipid variables (Figure S3) suggest most contain the n6 form of FA(20:4). The only relevant isoforms for FA(22:1) and FA(22:2) are 22:1n9 (erucic acid) and 22:2n6 (docosadienoic acid), respectively.

Figure S4 shows the biological relationships among the neutral and phospholipid lipid classes measured in the current study. LPC is a bioactive phospholipid synthesized primarily from plasma membrane- and lipoprotein-PC by phospholipase A1 (PLA1) or PLA2 that cleave the PC sn-1 or sn-2 ester bond, respectively [14,15,16]. LPC can also be formed by lecithin cholesterol acyltransferase in HDL, from oxidation of LDL and by endothelial lipase. LPC transports glycerophospholipid components between tissues but is also a ligand for specific signaling receptors and activates several second messengers [17]. Much experimental data have implicated LPC in atherosclerosis and acute and chronic inflammation but results support both beneficial and adverse properties [18,19]. The conflicting biological properties of LPC might be due to their fatty acyl composition, with saturated or monounsaturated LPC presenting with greater pro-atherogenic properties than polyunsaturated LPCs [20,21,22,23]. In the current study, 18 of the 20 LPCs examined tended to decrease with coffee intake but none of these shared a particular fatty acyl composition pattern (i.e., saturated or polyunsaturated fatty acids) (Table 1 and data not shown). LPC(20:4n6) sn-2, a potential isoform of LPC (20:4), is particularly interesting because it intersects several metabolic pathways that lead to the production of potent signaling molecules such as 2-arachidonoyl-lysophosphatidic acid, and specific eicosanoids and endocannabinoids [24,25,26]. Metabolites of the endocannabinoid system as well as choline (a product of LPC metabolism) and glycerol-3-phosphate (a product of LPC metabolism and endocannabinoid synthesis) significantly decreased in response to coffee intake in our previous metabolomic study [8,18,27]. Interestingly, PLA2 also contributes to endocannabinoid synthesis [27]. Taken together, decreased LPC levels in response to coffee align with decreased levels of downstream metabolites in similar biological pathways, most notably glycerophospholipid metabolism.

Although the caffeine component of coffee is known to stimulate lipolysis in the acute setting [28,29,30,31], the mechanisms and constituents of habitual coffee drinking leading to decreased LPC in the current study are unclear. The resistance of LDL to oxidative damage (a source of LPC) in humans increases after consumption of coffee and this might be explained by the incorporation of coffee’s phenolic acids into LDL [32]. Indeed, polyphenols, including those in coffee, decreased LPC production induced by oxidation [33].

Population-based or cross-sectional metabolomic/lipidomic studies of self-reported habitual coffee intake have also reported specific lipid species associated with coffee intake (Table S5). Direct comparison with the current report is difficult given the study designs, different lipidomic platforms used, and limitations in lipid species quantification (i.e., detected signals are usually a sum of several isobaric/isomeric lipids). Interestingly, however, Miranda et al. [34] focused exclusively on LPC species and found an inverse association between the plasma levels of LPC(16:1 a), LPC(18:1 a) and LPC(20:4 a) and habitual coffee intake, particularly when comparing intakes > 100 mL/day to 0 mL/day. 

The uncertain biological implications of lower LPC levels also extends to human studies of diseases or conditions of which are potentially modified by coffee consumption (Table S6). Most are cross-sectional and include small sample sizes and generate some significant findings that are not confirmed in other studies. Applicable to the original motivation of the data from our coffee trial examined here is a recent meta-analysis of metabolite changes and risk of T2D [35]; only three lipid markers were significantly associated with risk: LPC(18:0), SM(16:0) and FFA(18:1) [35]. None of these lipid species changed in response to coffee in the current study.

All lipid species that potentially increased in response to coffee did so only after the period of 8 cups per day. These lipid species also tended to correlate among each other rather than with lipid species that decreased in response to coffee, suggesting distinct lipid pathways altered by low and high coffee intake. PC species were a notable exception. PC species that increased in response to 8 cups of coffee all contained FA(18:0) and their changes directly correlated with changes in LPCs, TAGs, and acylcholines that decreased in response to 4 and 8 cups. This might suggest a shared lipid pathway impacted by coffee intake and the increase in PC observed only after 8 cups per day is a delayed dose response. 

In the initial report of the current coffee trial, several clinical lipid and inflammatory biomarkers changed in response to coffee [9]. None of these were convincingly correlated with lipids or metabolites measured in the current or recent report and underscores additional information accessible via high-throughput or more precise omic analysis. Triglycerides, for example, did not significantly change in the trial, yet when analyzing TAG species we observed TAGs that potentially increased and decreased in response to coffee. Nevertheless, a special complication in the analysis and clinical interpretation of TAGs is the large number of isobars resulting from presence of different combinations of the three acyl moieties and their regioisomers.

The application of lipidomics to a clinical study of coffee intake with repeated measures, large contrasts in coffee intake, excellent participant compliance and standardized protocols for sample handling and storage are major strengths of the current study. As a clinical trial it addresses many of the limitations of observational studies. In addition, the composition of brewed coffee varies as a function of bean type, roast and preparation methods; factors for which detailed information is rarely collected in population-based studies of coffee. Participants of our clinical trial were all provided the same coffee: a medium roast, 100% Arabica blend of Brazilian, Columbian, Central American and African coffee which is a popular type of coffee in Finland. Despite these strengths, several weaknesses of the study should be acknowledged. Our one-group study design without randomization, lack of blinding of participants and placebo control were limitations. We cannot rule out an impact of time-varying factors that may induce significant associations due to correlations with coffee. No specific guidelines were provided on coffee additives (i.e., sugar, cream) or beverages to consume in the place of coffee during the month of coffee abstinence. The very low levels of xanthine metabolites in the first month suggest participants largely refrained from consuming any caffeine-containing beverages [8,9]. Our previous report presented no obvious overlap with potential metabolite markers of dairy or tea consumption or lifestyle factors [8]. Body weight, a proxy for energy balance, remained stable throughout the trial. All participants for the current study were Finnish habitual coffee drinkers at increased risk of T2D which may limit the generalizability of our findings to other groups.

## 5. Conclusions

Our study provides the first thorough analysis of the lipidomic changes in response to controlled coffee consumption. The new findings suggest coffee alters glycerophospholipid metabolism and build on our previous metabolomic results that yield novel candidate pathways that offer insight to the mechanisms by which coffee may be exerting its health effects.

## Figures and Tables

**Table 1:** Significant lipid markers of coffee consumption *.

Lipid Class †	Lipid Species	Group Effect		Fold of Change §		
		*p*-Value	*q*-Value	4 Cups/0 Cup	8 Cups/0 Cup	8 Cups/4 Cups
CE	CE(20:4)	0.0296	0.4529	0.9	0.92	1.02
FFA	FFA(20:3)	0.0021	0.297	0.9	0.87	0.96
	FFA(20:4)	0.0012	0.2492	0.95	0.87	0.91
	FFA(22:2)	0.0481	0.4529	0.95	0.89	0.94
	FFA(22:6)	0.0415	0.4529	0.98	0.89	0.91
TAG	TAG47:1-FA17:0	0.0483	0.4529	1.26	1.4	1.11
	TAG51:3-FA15:0	0.0401	0.4529	0.82	0.91	1.11
	TAG52:4-FA16:1	0.0317	0.4529	0.8	0.92	1.15
	TAG52:5-FA16:1	0.0329	0.4529	0.77	0.89	1.16
	TAG52:5-FA20:5	0.05	0.4529	1.07	1.25	1.18
	TAG52:6-FA16:1	0.041	0.4529	0.78	0.9	1.14
	TAG53:3-FA16:0	0.0211	0.4529	0.88	0.87	1
	TAG53:3-FA18:1	0.0242	0.4529	0.9	0.93	1.03
	TAG53:4-FA16:0	0.0229	0.4529	0.84	0.89	1.07
	TAG53:4-FA18:2	0.0289	0.4529	0.82	0.88	1.08
	TAG53:5-FA18:3	0.048	0.4529	0.87	0.92	1.06
	TAG54:3-FA18:1	0.0354	0.4529	0.82	0.9	1.09
	TAG54:3-FA20:1	0.0368	0.4529	0.84	0.94	1.13
	TAG54:4-FA20:1	0.0306	0.4529	0.82	0.94	1.14
	TAG55:3-FA18:1	0.0353	0.4529	0.82	0.86	1.05
	TAG55:4-FA18:1	0.0198	0.4529	0.82	0.85	1.04
	TAG55:5-FA18:1	0.0208	0.4529	0.77	0.83	1.08
	TAG56:3-FA18:1	0.0103	0.4529	0.81	0.87	1.07
	TAG56:3-FA20:1	0.0155	0.4529	0.79	0.86	1.09
	TAG56:4-FA18:1	0.0124	0.4529	0.8	0.87	1.08
	TAG56:4-FA20:1	0.0314	0.4529	0.71	0.81	1.14
	TAG56:4-FA20:2	0.0141	0.4529	0.84	0.88	1.05
	TAG56:5-FA18:1	0.0221	0.4529	0.83	0.9	1.09
	TAG56:5-FA20:2	0.0051	0.4529	0.77	0.84	1.08
	TAG56:5-FA20:3	0.0215	0.4529	0.83	0.89	1.08
	TAG56:5-FA20:4	0.0447	0.4529	0.84	0.91	1.07
	TAG56:6-FA18:2	0.0132	0.4529	0.77	0.88	1.13
	TAG56:6-FA20:2	0.0206	0.4529	0.76	0.84	1.11
	TAG56:6-FA20:3	0.0077	0.4529	0.77	0.85	1.1
	TAG56:6-FA20:4	0.0306	0.4529	0.81	0.88	1.08
	TAG56:7-FA18:2	0.0457	0.4529	0.8	0.91	1.14
	TAG56:7-FA20:3	0.042	0.4529	0.79	0.85	1.07
	TAG56:7-FA22:4	0.0484	0.4529	0.87	0.92	1.06
	TAG56:7-FA22:5	0.0384	0.4529	0.85	0.95	1.12
	TAG56:9-FA20:4	0.0458	0.4529	0.83	0.92	1.11
	TAG56:9-FA22:6	0.0229	0.4529	0.85	0.92	1.08
	TAG57:8-FA22:6	0.0093	0.4529	0.87	0.91	1.04
	TAG58:10-FA20:5	0.0161	0.4529	0.86	0.94	1.09
	TAG58:10-FA22:5	0.0391	0.4529	0.74	0.84	1.14
	TAG58:10-FA22:6	0.0388	0.4529	0.72	0.8	1.11
	TAG58:7-FA22:4	0.0294	0.4529	0.81	0.89	1.11
	TAG58:7-FA22:5	0.0109	0.4529	0.79	0.85	1.07
	TAG58:8-FA20:4	0.0324	0.4529	0.85	0.9	1.06
	TAG58:8-FA22:5	0.0386	0.4529	0.79	0.85	1.08
	TAG58:9-FA22:5	0.0478	0.4529	0.78	0.86	1.1
	TAG60:10-FA22:5	0.0349	0.4529	0.85	0.9	1.06
	TAG60:10-FA22:6	0.0357	0.4529	0.82	0.92	1.13
	TAG60:11-FA22:5	0.0038	0.4529	0.8	0.92	1.16
LPC	LPC(15:0)	0.0142	0.4529	0.95	0.92	0.97
	LPC(17:0)	0.0017	0.2886	0.96	0.9	0.93
	LPC(18:1)	0.0423	0.4529	0.98	0.93	0.95
	LPC(20:2)	0.0094	0.4529	0.95	0.89	0.93
	LPC(20:3)	0.0362	0.4529	0.94	0.91	0.96
	LPC(20:4)	<0.0001	0.0088	0.94	0.87	0.93
	LPC(22:1)	<0.0001	0.0313	0.91	0.78	0.86
	LPC(22:2)	<0.0001	0.0051	0.94	0.79	0.84
PC	PC(17:0/20:4)	0.0183	0.4529	0.91	0.91	1
	PC(18:0/16:1)	0.0274	0.4529	1.09	1.3	1.19
	PC(18:0/18:3)	0.0375	0.4529	1.13	1.24	1.1
	PC(18:0/20:2)	0.0143	0.4529	1	1.11	1.11
	PC(18:0/20:3)	0.0361	0.4529	0.96	1.08	1.12
	PC(18:1/20:4)	0.0152	0.4529	0.92	0.91	0.99
PE	PE(18:0/20:1)	0.0203	0.4529	0.97	1.12	1.16
	PE(O-16:0/18:2)	0.0301	0.4529	1.08	1.19	1.11
	PE(O-18:0/20:3)	0.0458	0.4529	0.98	1.12	1.15
	PE(P-16:0/18:2)	0.0246	0.4529	1.07	1.18	1.1
	PE(P-16:0/22:4)	0.025	0.4529	0.89	1.01	1.14
	PE(P-18:0/18:2)	0.0406	0.4529	1.04	1.15	1.1
DCER	DCER(24:0)	0.0475	0.4529	1	1.1	1.1
LCER	LCER(26:1)	0.0097	0.4529	0.95	1.08	1.13

CE: cholesteryl ester; FFA: free fatty acid; TAG: triacylglycerol; LPC: lysophosphatidylcholine; PC: phosphatidylcholine; PE: phosphatidylethanolamine; DCER: dihydroceramide; LCER: lactosylceramide. * Shown are results from RMA that meet nominal significance (*p* < 0.05, column 3). Bold-faced lipid species meet significance threshold of *p* < 0.05 (column 3) and *q* < 0.05 (column 4). † neutral lipids: CE, FFA, TAG; phospholipids: LPC, PC, PE; sphingolipids: DCER, LCER. § ANOVA contrasts: lipid levels that increase in response to coffee are shaded red (*p* < 0.05) or pink (0.05 < *p* < 0.10) and lipid levels that decrease are colored green (*p* < 0.05) or light green (0.05 < *p* < 0.10).

## Supplementary Materials

The following are available online at http://www.mdpi.com/2072-6643/10/12/1851/s1, Supplementary Notes, Figure S1: PCA and multilevel PCA analysis, Figure S2: Serum concentrations of lipid species nominally associated with changes in response to coffee intake, Figure S3: Pearson correlations of changes in variable (lipid/metabolite/biomarker) levels after a) 4 cups/d compared to 0 cups/d b) 8 cups/d compared to 0 cups/d and c) 8 cups/d compared to 4 cups/d, Figure S4: Lipid pathway including neutral and phospholipid lipid classes measured in the current study, Figure S5: Multilevel PCA, Table S1: Baseline characteristics of coffee trial participants (*N* = 47), Table S2: Lipid species excluded from the current analysis, Table S3: Lipids analyzed in the current study, Table S4: Metabolite markers of coffee response reported by Cornelis et al, 2016, Table S5: Population-based lipidomic studies of habitual coffee consumption, Table S6: Circulating lysophosphatidylcholines and coffee-implicated disease or conditions.

## References

[1] Reyes C.M., Cornelis M.C. (2018). Caffeine in the diet: Country-level consumption and guidelines. Nutrients.

[2] Cornelis M. (2014). Gene-coffee interactions and health. Curr. Nutr. Rep..

[3] Higdon J.V., Frei B. (2006). Coffee and health: A review of recent human research. Crit. Rev. Food Sci. Nutr..

[4] Cowan T.E., Palmnas M.S., Yang J., Bomhof M.R., Ardell K.L., Reimer R.A., Vogel H.J., Shearer J. (2014). Chronic coffee consumption in the diet-induced obese rat: Impact on gut microbiota and serum metabolomics. J. Nutr. Biochem..

[5] Fredholm B.B., Battig K., Holmen J., Nehlig A., Zvartau E.E. (1999). Actions of caffeine in the brain with special reference to factors that contribute to its widespread use. Pharmacol. Rev..

[6] Gilbert R.M., Spiller G.A. (1984). Caffeine consumption. The Methylxanthine Beverages and Foods: Chemistry, Consumption, and Health Effects.

[7] Cornelis M.C., Hu F.B. (2013). Systems epidemiology: A new direction in nutrition and metabolic disease research. Curr. Nutr. Rep..

[8] Cornelis M.C., Erlund I., Michelotti G.A., Herder C., Westerhuis J.A., Tuomilehto J. (2018). Metabolomic response to coffee consumption: Application to a three-stage clinical trial. J. Intern. Med..

[9] Kempf K., Herder C., Erlund I., Kolb H., Martin S., Carstensen M., Koenig W., Sundvall J., Bidel S., Kuha S. (2010). Effects of coffee consumption on subclinical inflammation and other risk factors for type 2 diabetes: A clinical trial. Am. J. Clin. Nutr..

[10] Stephenson D.J., Hoeferlin L.A., Chalfant C.E. (2017). Lipidomics in translational research and the clinical significance of lipid-based biomarkers. Transl. Res..

[11] Farnell D.J., Popat H., Richmond S. (2016). Multilevel principal component analysis (MPCA) in shape analysis: A feasibility study in medical and dental imaging. Comput. Methods Programs Biomed..

[12] Benjamini Y., Hochberg Y. (1995). Controlling the false ciscovery rate: A practical and powerful approach to multiple testing. J. R. Stat. Soc. Ser. B (Methodol.).

[13] Shannon P., Markiel A., Ozier O., Baliga N.S., Wang J.T., Ramage D., Amin N., Schwikowski B., Ideker T. (2003). Cytoscape: A software environment for integrated models of biomolecular interaction networks. Genome Res..

[14] Dennis E.A., Cao J., Hsu Y.-H., Magrioti V., Kokotos G. (2011). Phospholipase a2 enzymes: Physical structure, biological function, disease implication, chemical inhibition, and therapeutic intervention. Chem. Rev..

[15] Richmond G.S., Smith T.K. (2011). Phospholipases a(1). Int. J. Mol. Sci..

[16] Yamashita A., Hayashi Y., Nemoto-Sasaki Y., Ito M., Oka S., Tanikawa T., Waku K., Sugiura T. (2014). Acyltransferases and transacylases that determine the fatty acid composition of glycerolipids and the metabolism of bioactive lipid mediators in mammalian cells and model organisms. Prog. Lipid Res..

[17] Tomura H., Mogi C., Sato K., Okajima F. (2005). Proton-sensing and lysolipid-sensitive g-protein-coupled receptors: A novel type of multi-functional receptors. Cell Signal..

[18] Schmitz G., Ruebsaamen K. (2010). Metabolism and atherogenic disease association of lysophosphatidylcholine. Atherosclerosis.

[19] Matsumoto T., Kobayashi T., Kamata K. (2007). Role of lysophosphatidylcholine (LPC) in atherosclerosis. Curr. Med. Chem..

[20] Hung N.D., Sok D.-E., Kim M.R. (2012). Prevention of 1-palmitoyl lysophosphatidylcholine-induced inflammation by polyunsaturated acyl lysophosphatidylcholine. Inflamm. Res..

[21] Akerele O., Cheema S. (2015). Fatty acyl composition of lysophosphatidylcholine is important in atherosclerosis. Med. Hypotheses.

[22] Ojala P., Hirvonen T., Hermansson M., Somerharju P., Parkkinen J. (2007). Acyl chain-dependent effect of lysophosphatidylcholine on human neutrophils. J. Leukoc. Boil..

[23] Aiyar N., Disa J., Ao Z., Ju H., Nerurkar S., Willette R.N., Macphee C.H., Johns D.G., Douglas S.A. (2007). Lysophosphatidylcholine induces inflammatory activation of human coronary artery smooth muscle cells. Mol. Cell. Biochem..

[24] Pete M.J., Exton J.H. (1996). Purification of a lysophospholipase from bovine brain that selectively deacylates arachidonoyl-substituted lysophosphatidylcholine. J. Boil. Chem..

[25] Di Marzo V. (1998). ‘Endocannabinoids’ and other fatty acid derivatives with cannabimimetic properties: Biochemistry and possible physiopathological relevance. Biochim. Biophys. Acta (BBA)-Lipids Lipid Metab..

[26] Aoki J., Inoue A., Okudaira S. (2008). Two pathways for lysophosphatidic acid production. Biochim. Biophys. Acta (BBA)-Mol. Cell Boil. Lipids.

[27] Maccarrone M. (2017). Metabolism of the endocannabinoid anandamide: Open questions after 25 years. Front. Mol. Neurosci..

[28] Beaudoin M.-S., Robinson L.E., Graham T.E. (2011). An oral lipid challenge and acute intake of caffeinated coffee additively decrease glucose tolerance in healthy men. J. Nutr..

[29] Mougios V., Ring S., Petridou A., Nikolaidis M.G. (2003). Duration of coffee-and exercise-induced changes in the fatty acid profile of human serum. J. Appl. Physiol..

[30] Bellet S., Kershbaum A., Finck E.M. (1968). Response of free fatty acids to coffee and caffeine. Metabolism.

[31] Hodgson A.B., Randell R.K., Jeukendrup A.E. (2013). The metabolic and performance effects of caffeine compared to coffee during endurance exercise. PLoS ONE.

[32] Natella F., Nardini M., Belelli F., Scaccini C. (2007). Coffee drinking induces incorporation of phenolic acids into LDL and increases the resistance of LDL to ex vivo oxidation in humans. Am. J. Clin. Nutr..

[33] Cartron E., Carbonneau M.-A., Fouret G., Descomps B., Léger C.L. (2001). Specific antioxidant activity of caffeoyl derivatives and other natural phenolic compounds: LDL protection against oxidation and decrease in the proinflammatory lysophosphatidylcholine production. J. Nat. Prod..

[34] Miranda A.M., Carioca A.A.F., Steluti J., da Silva I., Fisberg R.M., Marchioni D.M. (2017). The effect of coffee intake on lysophosphatidylcholines: A targeted metabolomic approach. Clin. Nutr. (Edinburgh Scotland).

[35] Park J.-E., Lim H.R., Kim J.W., Shin K.-H. (2018). Metabolite changes in risk of type 2 diabetes mellitus in cohort studies: A systematic review and meta-analysis. Diabetes Res. Clin. Pract..

