# Supplementary material for: Lipidomic Response to Coffee Consumption
Source: Nutrients. 2018 Dec 1;10(12):1851. doi: 10.3390/nu10121851 (PMC6315510; doi:10.3390/nu10121851)
Supplement: Supplementary file 1 [file nutrients-10-01851-s001.pdf]

Supplementary Information

Lipidomic response to coffee consumption

Supplementary Notes

1. Coffee Trial

The coffee trial design and primary clinical results have been reported in detail previously[1]. Habitual coffee consumers <65 years of age, residing in Finland, free of T2D, but with an elevated risk of T2D (≥13 points in the Finnish diabetes risk score) were eligible for participation. Exclusion criteria were the use of blood glucose–lowering medication or drugs that interfere with glucose metabolism, a history of chronic diseases likely to interfere with study participation, a history of alcohol or drug abuse, and pregnancy or breastfeeding. Eligible participants received 0, 3, or 5 packages with 500 g coffee/mo (Juhla Mokka, Paulig Group, Finland) prior to the first, second and third stage of the trial, respectively. The participants brewed the coffee daily at home with their own coffee machine using paper filters. They were allowed to divide their daily coffee dose as it suited them best and to keep the brewed coffee in a thermos if it was impossible to prepare it freshly before drinking. Instructions pertaining to the weight/volume of coffee to use per cup or the addition of cream, milk or sugar, were not provided. During the first month, participants refrained from drinking coffee, whereas in the second month they consumed 4 cups coffee/d (1 cup = 150 mL) and in the third month 8 cups/d. No restrictions were made on the consumption of other caffeine sources throughout the study. Participants were advised to maintain their normal physical activities and diet during the trial. Body weight remained stable throughout the trial. Of the 49 participants recruited, 47 completed the trial. Baseline characteristics of these 47 participants are shown in **Table S1**. At the end of each treatment stage, serum samples were collected after an overnight fast (including coffee) for ≥8 h and stored at -80ºC. Analysts performing the biochemical analyses were blinded to the treatment stage of each participant. The trial was conducted in accordance with the Declaration of Helsinki (1964), as amended in South Africa (1996), and approved by Joint Authority for the Hospital District of Helsinki and Uusimaa Ethics Committee, Department of Medicine, Helsinki, Finland. Written informed consent was obtained from all participants. Trial registration: http://www.isrctn.com/ISRCTN12547806 .

2. Multivariate Analysis: Coffee Drinker Classification and Predictive Lipid-Screening

Multilevel partial least squares discriminant analysis (MPLSDA)[2] was performed to examine whether coffee consumption led to systemic lipid changes and which lipids were the most differentiating biomarkers across the

stages of the coffee trial as described previously[3]. Because repeated measure multivariate methods that model three or more levels simultaneously are prone to bias[4], we performed separate MPLSDA for each treatment comparison (4 cups vs 0 cups, 8 vs 0 and 8 vs 4)[2].

Upon visual inspection of the multilevel PCA score plots for each coffee stage comparison, no clear classification was evident when modeling all lipid species (**Figure S5**). Comparing the non-coffee period (0 cups/d) to 4 cups/d and to 8 cups/d, on average 42.1 (44.8%) and 20.3 (21.6%) samples, respectively, out of 94 were misclassified. CV was not warranted.

Table S1. Baseline characteristics of coffee trial participants (N=47)[1]

Characteristic	Value
age, years	54.0 (9.0)
female, n (%)	36 (77)
body mass index, kg/m^2	29.2 (4.6)
waist circumference, cm	98.1 (10.5)
systolic blood pressure, mm Hg	141 (15)
diastolic blood pressure, mm Hg	90 (9)
current smoker, n (%)	2 (4)
habitual coffee intake, 150-ml cups/d	4.0 (1.7)
tea consumers, n (%)	17 (36)

Data are mean (SD) unless otherwise noted

Table S2: Lipid species excluded from the current analysis

Lipid Class	Lipid
CER	CER(20:1)
DAG	DAG(12:0/18:2)
DAG	DAG(14:0/14:0)
DAG	DAG(14:0/20:4)
DAG	DAG(14:0/22:6)
DAG	DAG(16:1/20:4)
DCER	DCER(14:0)
DCER	DCER(22:1)
DCER	DCER(26:1)
HCER	HCER(14:0)
LPC	LPC(18:4)
LPE	LPE(14:0)
LPE	LPE(14:1)
LPE	LPE(18:4)
LPE	LPE(20:0)
LPE	LPE(22:4)
PC	PC(12:0/16:1)
PC	PC(12:0/18:4)
PC	PC(12:0/20:4)
PC	PC(14:0/14:0)
PC	PC(14:0/20:1)
PC	PC(14:0/22:1)
PC	PC(14:0/22:2)
PC	PC(14:0/22:4)
PC	PC(14:0/22:5)
PC	PC(14:0/22:6)
PC	PC(15:0/16:1)
PC	PC(15:0/20:3)
PC	PC(15:0/20:4)
PC	PC(15:0/22:5)
PC	PC(15:0/22:6)
PC	PC(16:0/14:1)
PC	PC(16:0/18:4)
PC	PC(17:0/16:1)
PC	PC(17:0/18:3)
PC	PC(17:0/20:5)
PC	PC(17:0/22:5)
PC	PC(17:0/22:6)
PC	PC(18:0/18:4)
PC	PC(18:0/22:1)
PC	PC(18:1/22:1)
PC	PC(18:1/22:2)
PC	PC(18:1/22:4)

PC	PC(18:1/22:5)
PC	PC(18:2/22:1)
PC	PC(18:2/22:4)
PC	PC(18:2/22:5)
PC	PC(18:2/22:6)
PC	PC(20:0/16:1)
PC	PC(20:0/18:3)
PC	PC(20:0/20:3)
PE	PE(16:0/16:1)
PE	PE(16:0/18:3)
PE	PE(16:0/20:1)
PE	PE(16:0/20:2)
PE	PE(16:0/20:5)
PE	PE(16:0/22:4)
PE	PE(17:0/18:1)
PE	PE(17:0/18:2)
PE	PE(17:0/20:2)
PE	PE(17:0/20:4)
PE	PE(17:0/22:4)
PE	PE(18:0/15:0)
PE	PE(18:0/16:0)
PE	PE(18:0/16:1)
PE	PE(18:0/17:0)
PE	PE(18:1/14:1)
PE	PE(18:1/18:3)
PE	PE(18:1/20:1)
PE	PE(18:1/20:2)
PE	PE(18:1/20:3)
PE	PE(18:1/20:5)
PE	PE(18:1/22:4)
PE	PE(18:1/22:5)
PE	PE(18:1/22:6)
PE	PE(18:2/14:1)
PE	PE(18:2/18:2)
PE	PE(18:2/18:3)
PE	PE(18:2/20:4)
PE	PE(O-16:0/16:0)
PE	PE(O-16:0/20:3)
PE	PE(O-16:0/20:5)
PE	PE(O-16:0/22:4)
PE	PE(O-18:0/16:1)
PE	PE(O-18:0/18:0)
PE	PE(O-18:0/18:3)
PE	PE(O-18:0/20:5)
PE	PE(O-18:0/22:2)
PE	PE(O-18:0/22:4)

PE	PE(O-18:0/22:5)
PE	PE(O-18:0/22:6)
PE	PE(P-14:0/18:1)
PE	PE(P-16:0/16:1)
PE	PE(P-16:0/18:0)
PE	PE(P-16:0/18:3)
PE	PE(P-16:0/20:1)
PE	PE(P-16:0/20:2)
PE	PE(P-16:1/18:1)
PE	PE(P-18:0/16:1)
PE	PE(P-18:0/18:0)
PE	PE(P-18:0/18:3)
PE	PE(P-18:0/20:2)
PE	PE(P-18:0/22:4)
PE	PE(P-18:1/16:1)
PE	PE(P-18:1/18:0)
PE	PE(P-18:1/18:3)
PE	PE(P-18:1/22:4)
PE	PE(P-18:1/22:5)
PE	PE(P-18:2/18:2)
PE	PE(P-18:2/20:4)
PE	PE(P-18:2/22:6)
PI	PI(16:0/18:3)
PI	PI(16:0/22:4)
PI	PI(16:0/22:5)
PI	PI(16:0/22:6)
PI	PI(18:0/20:5)
PI	PI(18:0/22:4)
PI	PI(18:0/22:6)
PI	PI(18:1/20:3)
PI	PI(18:2/18:2)

Table S3: Lipids analyzed in the current study

Analysis	Lipid Group	Lipid Class	Trait Type	Lipid	Unit
Primary	Neutral Lipids	CE	Lipid Species	CE(12:0)	uM or %mole
Primary	Neutral Lipids	CE	Lipid Species	CE(14:0)	uM or %mole
Primary	Neutral Lipids	CE	Lipid Species	CE(14:1)	uM or %mole
Primary	Neutral Lipids	CE	Lipid Species	CE(15:0)	uM or %mole
Primary	Neutral Lipids	CE	Lipid Species	CE(16:0)	uM or %mole
Primary	Neutral Lipids	CE	Lipid Species	CE(16:1)	uM or %mole
Primary	Neutral Lipids	CE	Lipid Species	CE(17:0)	uM or %mole
Primary	Neutral Lipids	CE	Lipid Species	CE(18:0)	uM or %mole
Primary	Neutral Lipids	CE	Lipid Species	CE(18:1)	uM or %mole
Primary	Neutral Lipids	CE	Lipid Species	CE(18:2)	uM or %mole
Primary	Neutral Lipids	CE	Lipid Species	CE(18:3)	uM or %mole
Primary	Neutral Lipids	CE	Lipid Species	CE(18:4)	uM or %mole
Primary	Neutral Lipids	CE	Lipid Species	CE(20:0)	uM or %mole
Primary	Neutral Lipids	CE	Lipid Species	CE(20:1)	uM or %mole
Primary	Neutral Lipids	CE	Lipid Species	CE(20:2)	uM or %mole
Primary	Neutral Lipids	CE	Lipid Species	CE(20:3)	uM or %mole
Primary	Neutral Lipids	CE	Lipid Species	CE(20:4)	uM or %mole
Primary	Neutral Lipids	CE	Lipid Species	CE(20:5)	uM or %mole
Primary	Neutral Lipids	CE	Lipid Species	CE(22:0)	uM or %mole
Primary	Neutral Lipids	CE	Lipid Species	CE(22:1)	uM or %mole
Primary	Neutral Lipids	CE	Lipid Species	CE(22:2)	uM or %mole
Primary	Neutral Lipids	CE	Lipid Species	CE(22:4)	uM or %mole
Primary	Neutral Lipids	CE	Lipid Species	CE(22:5)	uM or %mole
Primary	Neutral Lipids	CE	Lipid Species	CE(22:6)	uM or %mole
Primary	Neutral Lipids	CE	Lipid Species	CE(24:0)	uM or %mole
Primary	Neutral Lipids	CE	Lipid Species	CE(24:1)	uM or %mole
Primary	Neutral Lipids	DAG	Lipid Species	DAG(12:0/16:0)	uM or %mole
Primary	Neutral Lipids	DAG	Lipid Species	DAG(12:0/18:0)	uM or %mole
Primary	Neutral Lipids	DAG	Lipid Species	DAG(12:0/18:1)	uM or %mole
Primary	Neutral Lipids	DAG	Lipid Species	DAG(14:0/16:0)	uM or %mole
Primary	Neutral Lipids	DAG	Lipid Species	DAG(14:0/16:1)	uM or %mole
Primary	Neutral Lipids	DAG	Lipid Species	DAG(14:0/18:1)	uM or %mole
Primary	Neutral Lipids	DAG	Lipid Species	DAG(14:0/18:2)	uM or %mole
Primary	Neutral Lipids	DAG	Lipid Species	DAG(14:0/18:3)	uM or %mole
Primary	Neutral Lipids	DAG	Lipid Species	DAG(14:0/20:0)	uM or %mole
Primary	Neutral Lipids	DAG	Lipid Species	DAG(14:1/16:0)	uM or %mole
Primary	Neutral Lipids	DAG	Lipid Species	DAG(14:1/18:1)	uM or %mole
Primary	Neutral Lipids	DAG	Lipid Species	DAG(15:0/18:1)	uM or %mole
Primary	Neutral Lipids	DAG	Lipid Species	DAG(15:0/18:2)	uM or %mole
Primary	Neutral Lipids	DAG	Lipid Species	DAG(16:0/16:0)	uM or %mole
Primary	Neutral Lipids	DAG	Lipid Species	DAG(16:0/16:1)	uM or %mole

Primary	Neutral Lipids	DAG	Lipid Species	DAG(16:0/18:0)	uM or %mole
Primary	Neutral Lipids	DAG	Lipid Species	DAG(16:0/18:1)	uM or %mole
Primary	Neutral Lipids	DAG	Lipid Species	DAG(16:0/18:2)	uM or %mole
Primary	Neutral Lipids	DAG	Lipid Species	DAG(16:0/18:3)	uM or %mole
Primary	Neutral Lipids	DAG	Lipid Species	DAG(16:0/20:3)	uM or %mole
Primary	Neutral Lipids	DAG	Lipid Species	DAG(16:0/20:4)	uM or %mole
Primary	Neutral Lipids	DAG	Lipid Species	DAG(16:0/22:6)	uM or %mole
Primary	Neutral Lipids	DAG	Lipid Species	DAG(16:1/16:1)	uM or %mole
Primary	Neutral Lipids	DAG	Lipid Species	DAG(16:1/18:0)	uM or %mole
Primary	Neutral Lipids	DAG	Lipid Species	DAG(16:1/18:1)	uM or %mole
Primary	Neutral Lipids	DAG	Lipid Species	DAG(16:1/18:2)	uM or %mole
Primary	Neutral Lipids	DAG	Lipid Species	DAG(16:1/18:3)	uM or %mole
Primary	Neutral Lipids	DAG	Lipid Species	DAG(16:1/20:0)	uM or %mole
Primary	Neutral Lipids	DAG	Lipid Species	DAG(16:1/20:2)	uM or %mole
Primary	Neutral Lipids	DAG	Lipid Species	DAG(16:1/22:6)	uM or %mole
Primary	Neutral Lipids	DAG	Lipid Species	DAG(18:0/18:1)	uM or %mole
Primary	Neutral Lipids	DAG	Lipid Species	DAG(18:0/18:2)	uM or %mole
Primary	Neutral Lipids	DAG	Lipid Species	DAG(18:0/18:3)	uM or %mole
Primary	Neutral Lipids	DAG	Lipid Species	DAG(18:0/22:6)	uM or %mole
Primary	Neutral Lipids	DAG	Lipid Species	DAG(18:1/18:1)	uM or %mole
Primary	Neutral Lipids	DAG	Lipid Species	DAG(18:1/18:2)	uM or %mole
Primary	Neutral Lipids	DAG	Lipid Species	DAG(18:1/20:1)	uM or %mole
Primary	Neutral Lipids	DAG	Lipid Species	DAG(18:1/20:2)	uM or %mole
Primary	Neutral Lipids	DAG	Lipid Species	DAG(18:1/20:3)	uM or %mole
Primary	Neutral Lipids	DAG	Lipid Species	DAG(18:1/20:4)	uM or %mole
Primary	Neutral Lipids	DAG	Lipid Species	DAG(18:1/22:4)	uM or %mole
Primary	Neutral Lipids	DAG	Lipid Species	DAG(18:1/22:6)	uM or %mole
Primary	Neutral Lipids	DAG	Lipid Species	DAG(18:2/18:3)	uM or %mole
Primary	Neutral Lipids	DAG	Lipid Species	DAG(18:2/20:3)	uM or %mole
Primary	Neutral Lipids	DAG	Lipid Species	DAG(18:2/20:4)	uM or %mole
Primary	Neutral Lipids	DAG	Lipid Species	DAG(18:2/22:4)	uM or %mole
Primary	Neutral Lipids	DAG	Lipid Species	DAG(18:2/22:6)	uM or %mole
Primary	Neutral Lipids	DAG	Lipid Species	DAG(20:0/20:0)	uM or %mole
Primary	Neutral Lipids	FFA	Lipid Species	FFA(12:0)	uM or %mole
Primary	Neutral Lipids	FFA	Lipid Species	FFA(14:0)	uM or %mole
Primary	Neutral Lipids	FFA	Lipid Species	FFA(14:1)	uM or %mole
Primary	Neutral Lipids	FFA	Lipid Species	FFA(15:0)	uM or %mole
Primary	Neutral Lipids	FFA	Lipid Species	FFA(16:0)	uM or %mole
Primary	Neutral Lipids	FFA	Lipid Species	FFA(16:1)	uM or %mole
Primary	Neutral Lipids	FFA	Lipid Species	FFA(17:0)	uM or %mole
Primary	Neutral Lipids	FFA	Lipid Species	FFA(18:0)	uM or %mole
Primary	Neutral Lipids	FFA	Lipid Species	FFA(18:1)	uM or %mole
Primary	Neutral Lipids	FFA	Lipid Species	FFA(18:2)	uM or %mole
Primary	Neutral Lipids	FFA	Lipid Species	FFA(18:4)	uM or %mole

Primary	Neutral Lipids	FFA	Lipid Species	FFA(20:0)	uM or %mole
Primary	Neutral Lipids	FFA	Lipid Species	FFA(20:1)	uM or %mole
Primary	Neutral Lipids	FFA	Lipid Species	FFA(20:2)	uM or %mole
Primary	Neutral Lipids	FFA	Lipid Species	FFA(20:3)	uM or %mole
Primary	Neutral Lipids	FFA	Lipid Species	FFA(20:4)	uM or %mole
Primary	Neutral Lipids	FFA	Lipid Species	FFA(20:5)	uM or %mole
Primary	Neutral Lipids	FFA	Lipid Species	FFA(22:0)	uM or %mole
Primary	Neutral Lipids	FFA	Lipid Species	FFA(22:1)	uM or %mole
Primary	Neutral Lipids	FFA	Lipid Species	FFA(22:2)	uM or %mole
Primary	Neutral Lipids	FFA	Lipid Species	FFA(22:4)	uM or %mole
Primary	Neutral Lipids	FFA	Lipid Species	FFA(22:5)	uM or %mole
Primary	Neutral Lipids	FFA	Lipid Species	FFA(22:6)	uM or %mole
Primary	Neutral Lipids	FFA	Lipid Species	FFA(24:0)	uM or %mole
Primary	Neutral Lipids	FFA	Lipid Species	FFA(24:1)	uM or %mole
Primary	Neutral Lipids	TAG	Lipid Species	TAG36:0-FA12:0	uM or %mole
Primary	Neutral Lipids	TAG	Lipid Species	TAG38:0-FA12:0	uM or %mole
Primary	Neutral Lipids	TAG	Lipid Species	TAG40:0-FA12:0	uM or %mole
Primary	Neutral Lipids	TAG	Lipid Species	TAG40:0-FA14:0	uM or %mole
Primary	Neutral Lipids	TAG	Lipid Species	TAG40:0-FA16:0	uM or %mole
Primary	Neutral Lipids	TAG	Lipid Species	TAG42:0-FA12:0	uM or %mole
Primary	Neutral Lipids	TAG	Lipid Species	TAG42:0-FA14:0	uM or %mole
Primary	Neutral Lipids	TAG	Lipid Species	TAG42:0-FA16:0	uM or %mole
Primary	Neutral Lipids	TAG	Lipid Species	TAG42:1-FA12:0	uM or %mole
Primary	Neutral Lipids	TAG	Lipid Species	TAG42:1-FA14:0	uM or %mole
Primary	Neutral Lipids	TAG	Lipid Species	TAG42:1-FA16:0	uM or %mole
Primary	Neutral Lipids	TAG	Lipid Species	TAG42:1-FA16:1	uM or %mole
Primary	Neutral Lipids	TAG	Lipid Species	TAG42:1-FA18:1	uM or %mole
Primary	Neutral Lipids	TAG	Lipid Species	TAG42:2-FA12:0	uM or %mole
Primary	Neutral Lipids	TAG	Lipid Species	TAG42:2-FA18:2	uM or %mole
Primary	Neutral Lipids	TAG	Lipid Species	TAG44:0-FA12:0	uM or %mole
Primary	Neutral Lipids	TAG	Lipid Species	TAG44:0-FA14:0	uM or %mole
Primary	Neutral Lipids	TAG	Lipid Species	TAG44:0-FA16:0	uM or %mole
Primary	Neutral Lipids	TAG	Lipid Species	TAG44:0-FA18:0	uM or %mole
Primary	Neutral Lipids	TAG	Lipid Species	TAG44:1-FA12:0	uM or %mole
Primary	Neutral Lipids	TAG	Lipid Species	TAG44:1-FA14:0	uM or %mole
Primary	Neutral Lipids	TAG	Lipid Species	TAG44:1-FA14:1	uM or %mole
Primary	Neutral Lipids	TAG	Lipid Species	TAG44:1-FA16:0	uM or %mole
Primary	Neutral Lipids	TAG	Lipid Species	TAG44:1-FA16:1	uM or %mole
Primary	Neutral Lipids	TAG	Lipid Species	TAG44:1-FA18:1	uM or %mole
Primary	Neutral Lipids	TAG	Lipid Species	TAG44:2-FA12:0	uM or %mole
Primary	Neutral Lipids	TAG	Lipid Species	TAG44:2-FA14:0	uM or %mole
Primary	Neutral Lipids	TAG	Lipid Species	TAG44:2-FA16:0	uM or %mole
Primary	Neutral Lipids	TAG	Lipid Species	TAG44:2-FA16:1	uM or %mole
Primary	Neutral Lipids	TAG	Lipid Species	TAG44:2-FA18:1	uM or %mole

Primary	Neutral Lipids	TAG	Lipid Species	TAG44:2-FA18:2	uM or %mole
Primary	Neutral Lipids	TAG	Lipid Species	TAG44:3-FA18:2	uM or %mole
Primary	Neutral Lipids	TAG	Lipid Species	TAG45:0-FA14:0	uM or %mole
Primary	Neutral Lipids	TAG	Lipid Species	TAG45:0-FA15:0	uM or %mole
Primary	Neutral Lipids	TAG	Lipid Species	TAG45:0-FA16:0	uM or %mole
Primary	Neutral Lipids	TAG	Lipid Species	TAG45:1-FA15:0	uM or %mole
Primary	Neutral Lipids	TAG	Lipid Species	TAG45:1-FA16:0	uM or %mole
Primary	Neutral Lipids	TAG	Lipid Species	TAG45:1-FA18:1	uM or %mole
Primary	Neutral Lipids	TAG	Lipid Species	TAG46:0-FA12:0	uM or %mole
Primary	Neutral Lipids	TAG	Lipid Species	TAG46:0-FA14:0	uM or %mole
Primary	Neutral Lipids	TAG	Lipid Species	TAG46:0-FA16:0	uM or %mole
Primary	Neutral Lipids	TAG	Lipid Species	TAG46:0-FA18:0	uM or %mole
Primary	Neutral Lipids	TAG	Lipid Species	TAG46:1-FA12:0	uM or %mole
Primary	Neutral Lipids	TAG	Lipid Species	TAG46:1-FA14:0	uM or %mole
Primary	Neutral Lipids	TAG	Lipid Species	TAG46:1-FA14:1	uM or %mole
Primary	Neutral Lipids	TAG	Lipid Species	TAG46:1-FA16:0	uM or %mole
Primary	Neutral Lipids	TAG	Lipid Species	TAG46:1-FA16:1	uM or %mole
Primary	Neutral Lipids	TAG	Lipid Species	TAG46:1-FA18:0	uM or %mole
Primary	Neutral Lipids	TAG	Lipid Species	TAG46:1-FA18:1	uM or %mole
Primary	Neutral Lipids	TAG	Lipid Species	TAG46:2-FA12:0	uM or %mole
Primary	Neutral Lipids	TAG	Lipid Species	TAG46:2-FA14:0	uM or %mole
Primary	Neutral Lipids	TAG	Lipid Species	TAG46:2-FA14:1	uM or %mole
Primary	Neutral Lipids	TAG	Lipid Species	TAG46:2-FA16:0	uM or %mole
Primary	Neutral Lipids	TAG	Lipid Species	TAG46:2-FA16:1	uM or %mole
Primary	Neutral Lipids	TAG	Lipid Species	TAG46:2-FA18:1	uM or %mole
Primary	Neutral Lipids	TAG	Lipid Species	TAG46:2-FA18:2	uM or %mole
Primary	Neutral Lipids	TAG	Lipid Species	TAG46:3-FA12:0	uM or %mole
Primary	Neutral Lipids	TAG	Lipid Species	TAG46:3-FA14:0	uM or %mole
Primary	Neutral Lipids	TAG	Lipid Species	TAG46:3-FA14:1	uM or %mole
Primary	Neutral Lipids	TAG	Lipid Species	TAG46:3-FA16:0	uM or %mole
Primary	Neutral Lipids	TAG	Lipid Species	TAG46:3-FA16:1	uM or %mole
Primary	Neutral Lipids	TAG	Lipid Species	TAG46:3-FA18:1	uM or %mole
Primary	Neutral Lipids	TAG	Lipid Species	TAG46:3-FA18:2	uM or %mole
Primary	Neutral Lipids	TAG	Lipid Species	TAG46:3-FA18:3	uM or %mole
Primary	Neutral Lipids	TAG	Lipid Species	TAG46:4-FA18:2	uM or %mole
Primary	Neutral Lipids	TAG	Lipid Species	TAG47:0-FA14:0	uM or %mole
Primary	Neutral Lipids	TAG	Lipid Species	TAG47:0-FA15:0	uM or %mole
Primary	Neutral Lipids	TAG	Lipid Species	TAG47:0-FA16:0	uM or %mole
Primary	Neutral Lipids	TAG	Lipid Species	TAG47:0-FA17:0	uM or %mole
Primary	Neutral Lipids	TAG	Lipid Species	TAG47:1-FA14:0	uM or %mole
Primary	Neutral Lipids	TAG	Lipid Species	TAG47:1-FA15:0	uM or %mole
Primary	Neutral Lipids	TAG	Lipid Species	TAG47:1-FA16:0	uM or %mole
Primary	Neutral Lipids	TAG	Lipid Species	TAG47:1-FA16:1	uM or %mole
Primary	Neutral Lipids	TAG	Lipid Species	TAG47:1-FA17:0	uM or %mole

Primary	Neutral Lipids	TAG	Lipid Species	TAG47:1-FA18:1	uM or %mole
Primary	Neutral Lipids	TAG	Lipid Species	TAG47:2-FA14:0	uM or %mole
Primary	Neutral Lipids	TAG	Lipid Species	TAG47:2-FA15:0	uM or %mole
Primary	Neutral Lipids	TAG	Lipid Species	TAG47:2-FA16:1	uM or %mole
Primary	Neutral Lipids	TAG	Lipid Species	TAG47:2-FA18:1	uM or %mole
Primary	Neutral Lipids	TAG	Lipid Species	TAG47:2-FA18:2	uM or %mole
Primary	Neutral Lipids	TAG	Lipid Species	TAG48:0-FA14:0	uM or %mole
Primary	Neutral Lipids	TAG	Lipid Species	TAG48:0-FA16:0	uM or %mole
Primary	Neutral Lipids	TAG	Lipid Species	TAG48:0-FA18:0	uM or %mole
Primary	Neutral Lipids	TAG	Lipid Species	TAG48:1-FA12:0	uM or %mole
Primary	Neutral Lipids	TAG	Lipid Species	TAG48:1-FA14:0	uM or %mole
Primary	Neutral Lipids	TAG	Lipid Species	TAG48:1-FA14:1	uM or %mole
Primary	Neutral Lipids	TAG	Lipid Species	TAG48:1-FA16:0	uM or %mole
Primary	Neutral Lipids	TAG	Lipid Species	TAG48:1-FA16:1	uM or %mole
Primary	Neutral Lipids	TAG	Lipid Species	TAG48:1-FA18:0	uM or %mole
Primary	Neutral Lipids	TAG	Lipid Species	TAG48:1-FA18:1	uM or %mole
Primary	Neutral Lipids	TAG	Lipid Species	TAG48:2-FA12:0	uM or %mole
Primary	Neutral Lipids	TAG	Lipid Species	TAG48:2-FA14:0	uM or %mole
Primary	Neutral Lipids	TAG	Lipid Species	TAG48:2-FA14:1	uM or %mole
Primary	Neutral Lipids	TAG	Lipid Species	TAG48:2-FA16:0	uM or %mole
Primary	Neutral Lipids	TAG	Lipid Species	TAG48:2-FA16:1	uM or %mole
Primary	Neutral Lipids	TAG	Lipid Species	TAG48:2-FA18:0	uM or %mole
Primary	Neutral Lipids	TAG	Lipid Species	TAG48:2-FA18:1	uM or %mole
Primary	Neutral Lipids	TAG	Lipid Species	TAG48:2-FA18:2	uM or %mole
Primary	Neutral Lipids	TAG	Lipid Species	TAG48:3-FA12:0	uM or %mole
Primary	Neutral Lipids	TAG	Lipid Species	TAG48:3-FA14:0	uM or %mole
Primary	Neutral Lipids	TAG	Lipid Species	TAG48:3-FA14:1	uM or %mole
Primary	Neutral Lipids	TAG	Lipid Species	TAG48:3-FA16:0	uM or %mole
Primary	Neutral Lipids	TAG	Lipid Species	TAG48:3-FA16:1	uM or %mole
Primary	Neutral Lipids	TAG	Lipid Species	TAG48:3-FA18:1	uM or %mole
Primary	Neutral Lipids	TAG	Lipid Species	TAG48:3-FA18:2	uM or %mole
Primary	Neutral Lipids	TAG	Lipid Species	TAG48:3-FA18:3	uM or %mole
Primary	Neutral Lipids	TAG	Lipid Species	TAG48:4-FA12:0	uM or %mole
Primary	Neutral Lipids	TAG	Lipid Species	TAG48:4-FA14:0	uM or %mole
Primary	Neutral Lipids	TAG	Lipid Species	TAG48:4-FA14:1	uM or %mole
Primary	Neutral Lipids	TAG	Lipid Species	TAG48:4-FA16:0	uM or %mole
Primary	Neutral Lipids	TAG	Lipid Species	TAG48:4-FA16:1	uM or %mole
Primary	Neutral Lipids	TAG	Lipid Species	TAG48:4-FA18:1	uM or %mole
Primary	Neutral Lipids	TAG	Lipid Species	TAG48:4-FA18:2	uM or %mole
Primary	Neutral Lipids	TAG	Lipid Species	TAG48:4-FA18:3	uM or %mole
Primary	Neutral Lipids	TAG	Lipid Species	TAG48:4-FA20:4	uM or %mole
Primary	Neutral Lipids	TAG	Lipid Species	TAG48:5-FA18:2	uM or %mole
Primary	Neutral Lipids	TAG	Lipid Species	TAG48:5-FA18:3	uM or %mole
Primary	Neutral Lipids	TAG	Lipid Species	TAG49:0-FA15:0	uM or %mole

Primary	Neutral Lipids	TAG	Lipid Species	TAG49:0-FA16:0	uM or %mole
Primary	Neutral Lipids	TAG	Lipid Species	TAG49:0-FA17:0	uM or %mole
Primary	Neutral Lipids	TAG	Lipid Species	TAG49:0-FA18:0	uM or %mole
Primary	Neutral Lipids	TAG	Lipid Species	TAG49:1-FA14:0	uM or %mole
Primary	Neutral Lipids	TAG	Lipid Species	TAG49:1-FA15:0	uM or %mole
Primary	Neutral Lipids	TAG	Lipid Species	TAG49:1-FA16:0	uM or %mole
Primary	Neutral Lipids	TAG	Lipid Species	TAG49:1-FA16:1	uM or %mole
Primary	Neutral Lipids	TAG	Lipid Species	TAG49:1-FA17:0	uM or %mole
Primary	Neutral Lipids	TAG	Lipid Species	TAG49:1-FA18:1	uM or %mole
Primary	Neutral Lipids	TAG	Lipid Species	TAG49:2-FA14:0	uM or %mole
Primary	Neutral Lipids	TAG	Lipid Species	TAG49:2-FA15:0	uM or %mole
Primary	Neutral Lipids	TAG	Lipid Species	TAG49:2-FA16:0	uM or %mole
Primary	Neutral Lipids	TAG	Lipid Species	TAG49:2-FA16:1	uM or %mole
Primary	Neutral Lipids	TAG	Lipid Species	TAG49:2-FA17:0	uM or %mole
Primary	Neutral Lipids	TAG	Lipid Species	TAG49:2-FA18:1	uM or %mole
Primary	Neutral Lipids	TAG	Lipid Species	TAG49:2-FA18:2	uM or %mole
Primary	Neutral Lipids	TAG	Lipid Species	TAG49:3-FA15:0	uM or %mole
Primary	Neutral Lipids	TAG	Lipid Species	TAG49:3-FA16:0	uM or %mole
Primary	Neutral Lipids	TAG	Lipid Species	TAG49:3-FA16:1	uM or %mole
Primary	Neutral Lipids	TAG	Lipid Species	TAG49:3-FA18:2	uM or %mole
Primary	Neutral Lipids	TAG	Lipid Species	TAG49:3-FA18:3	uM or %mole
Primary	Neutral Lipids	TAG	Lipid Species	TAG50:0-FA14:0	uM or %mole
Primary	Neutral Lipids	TAG	Lipid Species	TAG50:0-FA16:0	uM or %mole
Primary	Neutral Lipids	TAG	Lipid Species	TAG50:0-FA18:0	uM or %mole
Primary	Neutral Lipids	TAG	Lipid Species	TAG50:1-FA14:0	uM or %mole
Primary	Neutral Lipids	TAG	Lipid Species	TAG50:1-FA16:0	uM or %mole
Primary	Neutral Lipids	TAG	Lipid Species	TAG50:1-FA16:1	uM or %mole
Primary	Neutral Lipids	TAG	Lipid Species	TAG50:1-FA18:0	uM or %mole
Primary	Neutral Lipids	TAG	Lipid Species	TAG50:1-FA18:1	uM or %mole
Primary	Neutral Lipids	TAG	Lipid Species	TAG50:1-FA20:1	uM or %mole
Primary	Neutral Lipids	TAG	Lipid Species	TAG50:2-FA14:0	uM or %mole
Primary	Neutral Lipids	TAG	Lipid Species	TAG50:2-FA14:1	uM or %mole
Primary	Neutral Lipids	TAG	Lipid Species	TAG50:2-FA16:0	uM or %mole
Primary	Neutral Lipids	TAG	Lipid Species	TAG50:2-FA16:1	uM or %mole
Primary	Neutral Lipids	TAG	Lipid Species	TAG50:2-FA18:0	uM or %mole
Primary	Neutral Lipids	TAG	Lipid Species	TAG50:2-FA18:1	uM or %mole
Primary	Neutral Lipids	TAG	Lipid Species	TAG50:2-FA18:2	uM or %mole
Primary	Neutral Lipids	TAG	Lipid Species	TAG50:2-FA20:2	uM or %mole
Primary	Neutral Lipids	TAG	Lipid Species	TAG50:3-FA14:0	uM or %mole
Primary	Neutral Lipids	TAG	Lipid Species	TAG50:3-FA14:1	uM or %mole
Primary	Neutral Lipids	TAG	Lipid Species	TAG50:3-FA16:0	uM or %mole
Primary	Neutral Lipids	TAG	Lipid Species	TAG50:3-FA16:1	uM or %mole
Primary	Neutral Lipids	TAG	Lipid Species	TAG50:3-FA18:0	uM or %mole
Primary	Neutral Lipids	TAG	Lipid Species	TAG50:3-FA18:1	uM or %mole

Primary	Neutral Lipids	TAG	Lipid Species	TAG50:3-FA18:2	uM or %mole
Primary	Neutral Lipids	TAG	Lipid Species	TAG50:3-FA18:3	uM or %mole
Primary	Neutral Lipids	TAG	Lipid Species	TAG50:3-FA20:3	uM or %mole
Primary	Neutral Lipids	TAG	Lipid Species	TAG50:4-FA14:0	uM or %mole
Primary	Neutral Lipids	TAG	Lipid Species	TAG50:4-FA14:1	uM or %mole
Primary	Neutral Lipids	TAG	Lipid Species	TAG50:4-FA16:0	uM or %mole
Primary	Neutral Lipids	TAG	Lipid Species	TAG50:4-FA16:1	uM or %mole
Primary	Neutral Lipids	TAG	Lipid Species	TAG50:4-FA18:1	uM or %mole
Primary	Neutral Lipids	TAG	Lipid Species	TAG50:4-FA18:2	uM or %mole
Primary	Neutral Lipids	TAG	Lipid Species	TAG50:4-FA18:3	uM or %mole
Primary	Neutral Lipids	TAG	Lipid Species	TAG50:4-FA20:3	uM or %mole
Primary	Neutral Lipids	TAG	Lipid Species	TAG50:4-FA20:4	uM or %mole
Primary	Neutral Lipids	TAG	Lipid Species	TAG50:5-FA14:0	uM or %mole
Primary	Neutral Lipids	TAG	Lipid Species	TAG50:5-FA14:1	uM or %mole
Primary	Neutral Lipids	TAG	Lipid Species	TAG50:5-FA16:0	uM or %mole
Primary	Neutral Lipids	TAG	Lipid Species	TAG50:5-FA16:1	uM or %mole
Primary	Neutral Lipids	TAG	Lipid Species	TAG50:5-FA18:1	uM or %mole
Primary	Neutral Lipids	TAG	Lipid Species	TAG50:5-FA18:2	uM or %mole
Primary	Neutral Lipids	TAG	Lipid Species	TAG50:5-FA18:3	uM or %mole
Primary	Neutral Lipids	TAG	Lipid Species	TAG50:5-FA20:4	uM or %mole
Primary	Neutral Lipids	TAG	Lipid Species	TAG50:5-FA20:5	uM or %mole
Primary	Neutral Lipids	TAG	Lipid Species	TAG50:6-FA20:4	uM or %mole
Primary	Neutral Lipids	TAG	Lipid Species	TAG51:0-FA16:0	uM or %mole
Primary	Neutral Lipids	TAG	Lipid Species	TAG51:0-FA17:0	uM or %mole
Primary	Neutral Lipids	TAG	Lipid Species	TAG51:0-FA18:0	uM or %mole
Primary	Neutral Lipids	TAG	Lipid Species	TAG51:1-FA15:0	uM or %mole
Primary	Neutral Lipids	TAG	Lipid Species	TAG51:1-FA16:0	uM or %mole
Primary	Neutral Lipids	TAG	Lipid Species	TAG51:1-FA17:0	uM or %mole
Primary	Neutral Lipids	TAG	Lipid Species	TAG51:1-FA18:0	uM or %mole
Primary	Neutral Lipids	TAG	Lipid Species	TAG51:1-FA18:1	uM or %mole
Primary	Neutral Lipids	TAG	Lipid Species	TAG51:2-FA15:0	uM or %mole
Primary	Neutral Lipids	TAG	Lipid Species	TAG51:2-FA16:0	uM or %mole
Primary	Neutral Lipids	TAG	Lipid Species	TAG51:2-FA16:1	uM or %mole
Primary	Neutral Lipids	TAG	Lipid Species	TAG51:2-FA17:0	uM or %mole
Primary	Neutral Lipids	TAG	Lipid Species	TAG51:2-FA18:0	uM or %mole
Primary	Neutral Lipids	TAG	Lipid Species	TAG51:2-FA18:1	uM or %mole
Primary	Neutral Lipids	TAG	Lipid Species	TAG51:2-FA18:2	uM or %mole
Primary	Neutral Lipids	TAG	Lipid Species	TAG51:3-FA15:0	uM or %mole
Primary	Neutral Lipids	TAG	Lipid Species	TAG51:3-FA16:0	uM or %mole
Primary	Neutral Lipids	TAG	Lipid Species	TAG51:3-FA16:1	uM or %mole
Primary	Neutral Lipids	TAG	Lipid Species	TAG51:3-FA17:0	uM or %mole
Primary	Neutral Lipids	TAG	Lipid Species	TAG51:3-FA18:1	uM or %mole
Primary	Neutral Lipids	TAG	Lipid Species	TAG51:3-FA18:2	uM or %mole
Primary	Neutral Lipids	TAG	Lipid Species	TAG51:3-FA18:3	uM or %mole

Primary	Neutral Lipids	TAG	Lipid Species	TAG51:4-FA15:0	uM or %mole
Primary	Neutral Lipids	TAG	Lipid Species	TAG51:4-FA16:0	uM or %mole
Primary	Neutral Lipids	TAG	Lipid Species	TAG51:4-FA16:1	uM or %mole
Primary	Neutral Lipids	TAG	Lipid Species	TAG51:4-FA18:1	uM or %mole
Primary	Neutral Lipids	TAG	Lipid Species	TAG51:4-FA18:2	uM or %mole
Primary	Neutral Lipids	TAG	Lipid Species	TAG51:4-FA18:3	uM or %mole
Primary	Neutral Lipids	TAG	Lipid Species	TAG51:4-FA20:4	uM or %mole
Primary	Neutral Lipids	TAG	Lipid Species	TAG51:5-FA18:2	uM or %mole
Primary	Neutral Lipids	TAG	Lipid Species	TAG51:5-FA18:3	uM or %mole
Primary	Neutral Lipids	TAG	Lipid Species	TAG52:0-FA16:0	uM or %mole
Primary	Neutral Lipids	TAG	Lipid Species	TAG52:0-FA18:0	uM or %mole
Primary	Neutral Lipids	TAG	Lipid Species	TAG52:0-FA20:0	uM or %mole
Primary	Neutral Lipids	TAG	Lipid Species	TAG52:1-FA16:0	uM or %mole
Primary	Neutral Lipids	TAG	Lipid Species	TAG52:1-FA16:1	uM or %mole
Primary	Neutral Lipids	TAG	Lipid Species	TAG52:1-FA18:0	uM or %mole
Primary	Neutral Lipids	TAG	Lipid Species	TAG52:1-FA18:1	uM or %mole
Primary	Neutral Lipids	TAG	Lipid Species	TAG52:1-FA20:0	uM or %mole
Primary	Neutral Lipids	TAG	Lipid Species	TAG52:1-FA20:1	uM or %mole
Primary	Neutral Lipids	TAG	Lipid Species	TAG52:2-FA14:0	uM or %mole
Primary	Neutral Lipids	TAG	Lipid Species	TAG52:2-FA16:0	uM or %mole
Primary	Neutral Lipids	TAG	Lipid Species	TAG52:2-FA16:1	uM or %mole
Primary	Neutral Lipids	TAG	Lipid Species	TAG52:2-FA18:0	uM or %mole
Primary	Neutral Lipids	TAG	Lipid Species	TAG52:2-FA18:1	uM or %mole
Primary	Neutral Lipids	TAG	Lipid Species	TAG52:2-FA18:2	uM or %mole
Primary	Neutral Lipids	TAG	Lipid Species	TAG52:2-FA20:0	uM or %mole
Primary	Neutral Lipids	TAG	Lipid Species	TAG52:2-FA20:1	uM or %mole
Primary	Neutral Lipids	TAG	Lipid Species	TAG52:2-FA20:2	uM or %mole
Primary	Neutral Lipids	TAG	Lipid Species	TAG52:3-FA14:0	uM or %mole
Primary	Neutral Lipids	TAG	Lipid Species	TAG52:3-FA16:0	uM or %mole
Primary	Neutral Lipids	TAG	Lipid Species	TAG52:3-FA16:1	uM or %mole
Primary	Neutral Lipids	TAG	Lipid Species	TAG52:3-FA18:0	uM or %mole
Primary	Neutral Lipids	TAG	Lipid Species	TAG52:3-FA18:1	uM or %mole
Primary	Neutral Lipids	TAG	Lipid Species	TAG52:3-FA18:2	uM or %mole
Primary	Neutral Lipids	TAG	Lipid Species	TAG52:3-FA18:3	uM or %mole
Primary	Neutral Lipids	TAG	Lipid Species	TAG52:3-FA20:0	uM or %mole
Primary	Neutral Lipids	TAG	Lipid Species	TAG52:3-FA20:1	uM or %mole
Primary	Neutral Lipids	TAG	Lipid Species	TAG52:3-FA20:2	uM or %mole
Primary	Neutral Lipids	TAG	Lipid Species	TAG52:3-FA20:3	uM or %mole
Primary	Neutral Lipids	TAG	Lipid Species	TAG52:3-FA22:1	uM or %mole
Primary	Neutral Lipids	TAG	Lipid Species	TAG52:4-FA14:0	uM or %mole
Primary	Neutral Lipids	TAG	Lipid Species	TAG52:4-FA16:0	uM or %mole
Primary	Neutral Lipids	TAG	Lipid Species	TAG52:4-FA16:1	uM or %mole
Primary	Neutral Lipids	TAG	Lipid Species	TAG52:4-FA18:0	uM or %mole
Primary	Neutral Lipids	TAG	Lipid Species	TAG52:4-FA18:1	uM or %mole

Primary	Neutral Lipids	TAG	Lipid Species	TAG52:4-FA18:2	uM or %mole
Primary	Neutral Lipids	TAG	Lipid Species	TAG52:4-FA18:3	uM or %mole
Primary	Neutral Lipids	TAG	Lipid Species	TAG52:4-FA20:0	uM or %mole
Primary	Neutral Lipids	TAG	Lipid Species	TAG52:4-FA20:2	uM or %mole
Primary	Neutral Lipids	TAG	Lipid Species	TAG52:4-FA20:3	uM or %mole
Primary	Neutral Lipids	TAG	Lipid Species	TAG52:4-FA20:4	uM or %mole
Primary	Neutral Lipids	TAG	Lipid Species	TAG52:4-FA22:1	uM or %mole
Primary	Neutral Lipids	TAG	Lipid Species	TAG52:4-FA22:4	uM or %mole
Primary	Neutral Lipids	TAG	Lipid Species	TAG52:5-FA14:0	uM or %mole
Primary	Neutral Lipids	TAG	Lipid Species	TAG52:5-FA16:0	uM or %mole
Primary	Neutral Lipids	TAG	Lipid Species	TAG52:5-FA16:1	uM or %mole
Primary	Neutral Lipids	TAG	Lipid Species	TAG52:5-FA18:1	uM or %mole
Primary	Neutral Lipids	TAG	Lipid Species	TAG52:5-FA18:2	uM or %mole
Primary	Neutral Lipids	TAG	Lipid Species	TAG52:5-FA18:3	uM or %mole
Primary	Neutral Lipids	TAG	Lipid Species	TAG52:5-FA20:3	uM or %mole
Primary	Neutral Lipids	TAG	Lipid Species	TAG52:5-FA20:4	uM or %mole
Primary	Neutral Lipids	TAG	Lipid Species	TAG52:5-FA20:5	uM or %mole
Primary	Neutral Lipids	TAG	Lipid Species	TAG52:5-FA22:5	uM or %mole
Primary	Neutral Lipids	TAG	Lipid Species	TAG52:6-FA14:0	uM or %mole
Primary	Neutral Lipids	TAG	Lipid Species	TAG52:6-FA16:0	uM or %mole
Primary	Neutral Lipids	TAG	Lipid Species	TAG52:6-FA16:1	uM or %mole
Primary	Neutral Lipids	TAG	Lipid Species	TAG52:6-FA18:1	uM or %mole
Primary	Neutral Lipids	TAG	Lipid Species	TAG52:6-FA18:2	uM or %mole
Primary	Neutral Lipids	TAG	Lipid Species	TAG52:6-FA18:3	uM or %mole
Primary	Neutral Lipids	TAG	Lipid Species	TAG52:6-FA20:4	uM or %mole
Primary	Neutral Lipids	TAG	Lipid Species	TAG52:6-FA20:5	uM or %mole
Primary	Neutral Lipids	TAG	Lipid Species	TAG52:6-FA22:6	uM or %mole
Primary	Neutral Lipids	TAG	Lipid Species	TAG52:7-FA16:0	uM or %mole
Primary	Neutral Lipids	TAG	Lipid Species	TAG52:7-FA18:1	uM or %mole
Primary	Neutral Lipids	TAG	Lipid Species	TAG52:7-FA20:5	uM or %mole
Primary	Neutral Lipids	TAG	Lipid Species	TAG52:7-FA22:6	uM or %mole
Primary	Neutral Lipids	TAG	Lipid Species	TAG52:8-FA16:1	uM or %mole
Primary	Neutral Lipids	TAG	Lipid Species	TAG52:8-FA18:2	uM or %mole
Primary	Neutral Lipids	TAG	Lipid Species	TAG53:0-FA16:0	uM or %mole
Primary	Neutral Lipids	TAG	Lipid Species	TAG53:1-FA16:0	uM or %mole
Primary	Neutral Lipids	TAG	Lipid Species	TAG53:1-FA17:0	uM or %mole
Primary	Neutral Lipids	TAG	Lipid Species	TAG53:1-FA18:0	uM or %mole
Primary	Neutral Lipids	TAG	Lipid Species	TAG53:1-FA18:1	uM or %mole
Primary	Neutral Lipids	TAG	Lipid Species	TAG53:2-FA16:0	uM or %mole
Primary	Neutral Lipids	TAG	Lipid Species	TAG53:2-FA17:0	uM or %mole
Primary	Neutral Lipids	TAG	Lipid Species	TAG53:2-FA18:0	uM or %mole
Primary	Neutral Lipids	TAG	Lipid Species	TAG53:2-FA18:1	uM or %mole
Primary	Neutral Lipids	TAG	Lipid Species	TAG53:2-FA18:2	uM or %mole
Primary	Neutral Lipids	TAG	Lipid Species	TAG53:3-FA16:0	uM or %mole

Primary	Neutral Lipids	TAG	Lipid Species	TAG53:3-FA17:0	uM or %mole
Primary	Neutral Lipids	TAG	Lipid Species	TAG53:3-FA18:0	uM or %mole
Primary	Neutral Lipids	TAG	Lipid Species	TAG53:3-FA18:1	uM or %mole
Primary	Neutral Lipids	TAG	Lipid Species	TAG53:3-FA18:2	uM or %mole
Primary	Neutral Lipids	TAG	Lipid Species	TAG53:4-FA16:0	uM or %mole
Primary	Neutral Lipids	TAG	Lipid Species	TAG53:4-FA17:0	uM or %mole
Primary	Neutral Lipids	TAG	Lipid Species	TAG53:4-FA18:0	uM or %mole
Primary	Neutral Lipids	TAG	Lipid Species	TAG53:4-FA18:1	uM or %mole
Primary	Neutral Lipids	TAG	Lipid Species	TAG53:4-FA18:2	uM or %mole
Primary	Neutral Lipids	TAG	Lipid Species	TAG53:4-FA18:3	uM or %mole
Primary	Neutral Lipids	TAG	Lipid Species	TAG53:4-FA20:4	uM or %mole
Primary	Neutral Lipids	TAG	Lipid Species	TAG53:5-FA18:1	uM or %mole
Primary	Neutral Lipids	TAG	Lipid Species	TAG53:5-FA18:2	uM or %mole
Primary	Neutral Lipids	TAG	Lipid Species	TAG53:5-FA18:3	uM or %mole
Primary	Neutral Lipids	TAG	Lipid Species	TAG53:5-FA20:4	uM or %mole
Primary	Neutral Lipids	TAG	Lipid Species	TAG53:6-FA18:1	uM or %mole
Primary	Neutral Lipids	TAG	Lipid Species	TAG53:6-FA18:2	uM or %mole
Primary	Neutral Lipids	TAG	Lipid Species	TAG53:6-FA18:3	uM or %mole
Primary	Neutral Lipids	TAG	Lipid Species	TAG53:6-FA20:4	uM or %mole
Primary	Neutral Lipids	TAG	Lipid Species	TAG53:7-FA18:3	uM or %mole
Primary	Neutral Lipids	TAG	Lipid Species	TAG54:0-FA16:0	uM or %mole
Primary	Neutral Lipids	TAG	Lipid Species	TAG54:0-FA18:0	uM or %mole
Primary	Neutral Lipids	TAG	Lipid Species	TAG54:1-FA16:0	uM or %mole
Primary	Neutral Lipids	TAG	Lipid Species	TAG54:1-FA18:0	uM or %mole
Primary	Neutral Lipids	TAG	Lipid Species	TAG54:1-FA18:1	uM or %mole
Primary	Neutral Lipids	TAG	Lipid Species	TAG54:1-FA20:0	uM or %mole
Primary	Neutral Lipids	TAG	Lipid Species	TAG54:1-FA20:1	uM or %mole
Primary	Neutral Lipids	TAG	Lipid Species	TAG54:2-FA16:0	uM or %mole
Primary	Neutral Lipids	TAG	Lipid Species	TAG54:2-FA18:0	uM or %mole
Primary	Neutral Lipids	TAG	Lipid Species	TAG54:2-FA18:1	uM or %mole
Primary	Neutral Lipids	TAG	Lipid Species	TAG54:2-FA18:2	uM or %mole
Primary	Neutral Lipids	TAG	Lipid Species	TAG54:2-FA20:0	uM or %mole
Primary	Neutral Lipids	TAG	Lipid Species	TAG54:2-FA20:1	uM or %mole
Primary	Neutral Lipids	TAG	Lipid Species	TAG54:2-FA20:2	uM or %mole
Primary	Neutral Lipids	TAG	Lipid Species	TAG54:3-FA16:0	uM or %mole
Primary	Neutral Lipids	TAG	Lipid Species	TAG54:3-FA16:1	uM or %mole
Primary	Neutral Lipids	TAG	Lipid Species	TAG54:3-FA18:0	uM or %mole
Primary	Neutral Lipids	TAG	Lipid Species	TAG54:3-FA18:1	uM or %mole
Primary	Neutral Lipids	TAG	Lipid Species	TAG54:3-FA18:2	uM or %mole
Primary	Neutral Lipids	TAG	Lipid Species	TAG54:3-FA18:3	uM or %mole
Primary	Neutral Lipids	TAG	Lipid Species	TAG54:3-FA20:1	uM or %mole
Primary	Neutral Lipids	TAG	Lipid Species	TAG54:3-FA20:2	uM or %mole
Primary	Neutral Lipids	TAG	Lipid Species	TAG54:3-FA20:3	uM or %mole
Primary	Neutral Lipids	TAG	Lipid Species	TAG54:4-FA16:0	uM or %mole

Primary	Neutral Lipids	TAG	Lipid Species	TAG54:4-FA16:1	uM or %mole
Primary	Neutral Lipids	TAG	Lipid Species	TAG54:4-FA18:0	uM or %mole
Primary	Neutral Lipids	TAG	Lipid Species	TAG54:4-FA18:1	uM or %mole
Primary	Neutral Lipids	TAG	Lipid Species	TAG54:4-FA18:2	uM or %mole
Primary	Neutral Lipids	TAG	Lipid Species	TAG54:4-FA18:3	uM or %mole
Primary	Neutral Lipids	TAG	Lipid Species	TAG54:4-FA20:1	uM or %mole
Primary	Neutral Lipids	TAG	Lipid Species	TAG54:4-FA20:2	uM or %mole
Primary	Neutral Lipids	TAG	Lipid Species	TAG54:4-FA20:3	uM or %mole
Primary	Neutral Lipids	TAG	Lipid Species	TAG54:4-FA20:4	uM or %mole
Primary	Neutral Lipids	TAG	Lipid Species	TAG54:4-FA22:1	uM or %mole
Primary	Neutral Lipids	TAG	Lipid Species	TAG54:4-FA22:4	uM or %mole
Primary	Neutral Lipids	TAG	Lipid Species	TAG54:5-FA16:0	uM or %mole
Primary	Neutral Lipids	TAG	Lipid Species	TAG54:5-FA16:1	uM or %mole
Primary	Neutral Lipids	TAG	Lipid Species	TAG54:5-FA18:0	uM or %mole
Primary	Neutral Lipids	TAG	Lipid Species	TAG54:5-FA18:1	uM or %mole
Primary	Neutral Lipids	TAG	Lipid Species	TAG54:5-FA18:2	uM or %mole
Primary	Neutral Lipids	TAG	Lipid Species	TAG54:5-FA18:3	uM or %mole
Primary	Neutral Lipids	TAG	Lipid Species	TAG54:5-FA20:2	uM or %mole
Primary	Neutral Lipids	TAG	Lipid Species	TAG54:5-FA20:3	uM or %mole
Primary	Neutral Lipids	TAG	Lipid Species	TAG54:5-FA20:4	uM or %mole
Primary	Neutral Lipids	TAG	Lipid Species	TAG54:5-FA20:5	uM or %mole
Primary	Neutral Lipids	TAG	Lipid Species	TAG54:5-FA22:1	uM or %mole
Primary	Neutral Lipids	TAG	Lipid Species	TAG54:5-FA22:4	uM or %mole
Primary	Neutral Lipids	TAG	Lipid Species	TAG54:5-FA22:5	uM or %mole
Primary	Neutral Lipids	TAG	Lipid Species	TAG54:6-FA16:0	uM or %mole
Primary	Neutral Lipids	TAG	Lipid Species	TAG54:6-FA16:1	uM or %mole
Primary	Neutral Lipids	TAG	Lipid Species	TAG54:6-FA18:1	uM or %mole
Primary	Neutral Lipids	TAG	Lipid Species	TAG54:6-FA18:2	uM or %mole
Primary	Neutral Lipids	TAG	Lipid Species	TAG54:6-FA18:3	uM or %mole
Primary	Neutral Lipids	TAG	Lipid Species	TAG54:6-FA20:3	uM or %mole
Primary	Neutral Lipids	TAG	Lipid Species	TAG54:6-FA20:4	uM or %mole
Primary	Neutral Lipids	TAG	Lipid Species	TAG54:6-FA20:5	uM or %mole
Primary	Neutral Lipids	TAG	Lipid Species	TAG54:6-FA22:5	uM or %mole
Primary	Neutral Lipids	TAG	Lipid Species	TAG54:6-FA22:6	uM or %mole
Primary	Neutral Lipids	TAG	Lipid Species	TAG54:7-FA16:1	uM or %mole
Primary	Neutral Lipids	TAG	Lipid Species	TAG54:7-FA18:1	uM or %mole
Primary	Neutral Lipids	TAG	Lipid Species	TAG54:7-FA18:2	uM or %mole
Primary	Neutral Lipids	TAG	Lipid Species	TAG54:7-FA18:3	uM or %mole
Primary	Neutral Lipids	TAG	Lipid Species	TAG54:7-FA20:4	uM or %mole
Primary	Neutral Lipids	TAG	Lipid Species	TAG54:7-FA20:5	uM or %mole
Primary	Neutral Lipids	TAG	Lipid Species	TAG54:7-FA22:5	uM or %mole
Primary	Neutral Lipids	TAG	Lipid Species	TAG54:7-FA22:6	uM or %mole
Primary	Neutral Lipids	TAG	Lipid Species	TAG54:8-FA18:2	uM or %mole
Primary	Neutral Lipids	TAG	Lipid Species	TAG54:8-FA18:3	uM or %mole

Primary	Neutral Lipids	TAG	Lipid Species	TAG54:8-FA20:4	uM or %mole
Primary	Neutral Lipids	TAG	Lipid Species	TAG54:8-FA20:5	uM or %mole
Primary	Neutral Lipids	TAG	Lipid Species	TAG54:8-FA22:6	uM or %mole
Primary	Neutral Lipids	TAG	Lipid Species	TAG55:1-FA16:0	uM or %mole
Primary	Neutral Lipids	TAG	Lipid Species	TAG55:1-FA18:1	uM or %mole
Primary	Neutral Lipids	TAG	Lipid Species	TAG55:2-FA18:1	uM or %mole
Primary	Neutral Lipids	TAG	Lipid Species	TAG55:2-FA18:2	uM or %mole
Primary	Neutral Lipids	TAG	Lipid Species	TAG55:3-FA18:1	uM or %mole
Primary	Neutral Lipids	TAG	Lipid Species	TAG55:3-FA18:2	uM or %mole
Primary	Neutral Lipids	TAG	Lipid Species	TAG55:4-FA18:1	uM or %mole
Primary	Neutral Lipids	TAG	Lipid Species	TAG55:4-FA18:2	uM or %mole
Primary	Neutral Lipids	TAG	Lipid Species	TAG55:5-FA18:1	uM or %mole
Primary	Neutral Lipids	TAG	Lipid Species	TAG55:5-FA18:2	uM or %mole
Primary	Neutral Lipids	TAG	Lipid Species	TAG55:5-FA20:4	uM or %mole
Primary	Neutral Lipids	TAG	Lipid Species	TAG55:6-FA18:1	uM or %mole
Primary	Neutral Lipids	TAG	Lipid Species	TAG55:6-FA20:3	uM or %mole
Primary	Neutral Lipids	TAG	Lipid Species	TAG55:6-FA20:4	uM or %mole
Primary	Neutral Lipids	TAG	Lipid Species	TAG55:7-FA15:0	uM or %mole
Primary	Neutral Lipids	TAG	Lipid Species	TAG55:7-FA18:1	uM or %mole
Primary	Neutral Lipids	TAG	Lipid Species	TAG55:7-FA20:3	uM or %mole
Primary	Neutral Lipids	TAG	Lipid Species	TAG55:7-FA20:4	uM or %mole
Primary	Neutral Lipids	TAG	Lipid Species	TAG55:7-FA22:6	uM or %mole
Primary	Neutral Lipids	TAG	Lipid Species	TAG55:8-FA20:4	uM or %mole
Primary	Neutral Lipids	TAG	Lipid Species	TAG56:10-FA18:2	uM or %mole
Primary	Neutral Lipids	TAG	Lipid Species	TAG56:1-FA16:0	uM or %mole
Primary	Neutral Lipids	TAG	Lipid Species	TAG56:1-FA18:1	uM or %mole
Primary	Neutral Lipids	TAG	Lipid Species	TAG56:2-FA16:0	uM or %mole
Primary	Neutral Lipids	TAG	Lipid Species	TAG56:2-FA18:0	uM or %mole
Primary	Neutral Lipids	TAG	Lipid Species	TAG56:2-FA18:1	uM or %mole
Primary	Neutral Lipids	TAG	Lipid Species	TAG56:2-FA20:0	uM or %mole
Primary	Neutral Lipids	TAG	Lipid Species	TAG56:2-FA20:1	uM or %mole
Primary	Neutral Lipids	TAG	Lipid Species	TAG56:3-FA16:0	uM or %mole
Primary	Neutral Lipids	TAG	Lipid Species	TAG56:3-FA18:0	uM or %mole
Primary	Neutral Lipids	TAG	Lipid Species	TAG56:3-FA18:1	uM or %mole
Primary	Neutral Lipids	TAG	Lipid Species	TAG56:3-FA18:2	uM or %mole
Primary	Neutral Lipids	TAG	Lipid Species	TAG56:3-FA20:0	uM or %mole
Primary	Neutral Lipids	TAG	Lipid Species	TAG56:3-FA20:1	uM or %mole
Primary	Neutral Lipids	TAG	Lipid Species	TAG56:3-FA20:2	uM or %mole
Primary	Neutral Lipids	TAG	Lipid Species	TAG56:4-FA16:0	uM or %mole
Primary	Neutral Lipids	TAG	Lipid Species	TAG56:4-FA18:0	uM or %mole
Primary	Neutral Lipids	TAG	Lipid Species	TAG56:4-FA18:1	uM or %mole
Primary	Neutral Lipids	TAG	Lipid Species	TAG56:4-FA18:2	uM or %mole
Primary	Neutral Lipids	TAG	Lipid Species	TAG56:4-FA20:1	uM or %mole
Primary	Neutral Lipids	TAG	Lipid Species	TAG56:4-FA20:2	uM or %mole

Primary	Neutral Lipids	TAG	Lipid Species	TAG56:4-FA20:3	uM or %mole
Primary	Neutral Lipids	TAG	Lipid Species	TAG56:4-FA20:4	uM or %mole
Primary	Neutral Lipids	TAG	Lipid Species	TAG56:4-FA22:4	uM or %mole
Primary	Neutral Lipids	TAG	Lipid Species	TAG56:5-FA16:0	uM or %mole
Primary	Neutral Lipids	TAG	Lipid Species	TAG56:5-FA18:0	uM or %mole
Primary	Neutral Lipids	TAG	Lipid Species	TAG56:5-FA18:1	uM or %mole
Primary	Neutral Lipids	TAG	Lipid Species	TAG56:5-FA18:2	uM or %mole
Primary	Neutral Lipids	TAG	Lipid Species	TAG56:5-FA20:1	uM or %mole
Primary	Neutral Lipids	TAG	Lipid Species	TAG56:5-FA20:2	uM or %mole
Primary	Neutral Lipids	TAG	Lipid Species	TAG56:5-FA20:3	uM or %mole
Primary	Neutral Lipids	TAG	Lipid Species	TAG56:5-FA20:4	uM or %mole
Primary	Neutral Lipids	TAG	Lipid Species	TAG56:5-FA22:4	uM or %mole
Primary	Neutral Lipids	TAG	Lipid Species	TAG56:5-FA22:5	uM or %mole
Primary	Neutral Lipids	TAG	Lipid Species	TAG56:6-FA16:0	uM or %mole
Primary	Neutral Lipids	TAG	Lipid Species	TAG56:6-FA18:0	uM or %mole
Primary	Neutral Lipids	TAG	Lipid Species	TAG56:6-FA18:1	uM or %mole
Primary	Neutral Lipids	TAG	Lipid Species	TAG56:6-FA18:2	uM or %mole
Primary	Neutral Lipids	TAG	Lipid Species	TAG56:6-FA18:3	uM or %mole
Primary	Neutral Lipids	TAG	Lipid Species	TAG56:6-FA20:2	uM or %mole
Primary	Neutral Lipids	TAG	Lipid Species	TAG56:6-FA20:3	uM or %mole
Primary	Neutral Lipids	TAG	Lipid Species	TAG56:6-FA20:4	uM or %mole
Primary	Neutral Lipids	TAG	Lipid Species	TAG56:6-FA20:5	uM or %mole
Primary	Neutral Lipids	TAG	Lipid Species	TAG56:6-FA22:4	uM or %mole
Primary	Neutral Lipids	TAG	Lipid Species	TAG56:6-FA22:5	uM or %mole
Primary	Neutral Lipids	TAG	Lipid Species	TAG56:6-FA22:6	uM or %mole
Primary	Neutral Lipids	TAG	Lipid Species	TAG56:7-FA16:0	uM or %mole
Primary	Neutral Lipids	TAG	Lipid Species	TAG56:7-FA16:1	uM or %mole
Primary	Neutral Lipids	TAG	Lipid Species	TAG56:7-FA18:0	uM or %mole
Primary	Neutral Lipids	TAG	Lipid Species	TAG56:7-FA18:1	uM or %mole
Primary	Neutral Lipids	TAG	Lipid Species	TAG56:7-FA18:2	uM or %mole
Primary	Neutral Lipids	TAG	Lipid Species	TAG56:7-FA18:3	uM or %mole
Primary	Neutral Lipids	TAG	Lipid Species	TAG56:7-FA20:3	uM or %mole
Primary	Neutral Lipids	TAG	Lipid Species	TAG56:7-FA20:4	uM or %mole
Primary	Neutral Lipids	TAG	Lipid Species	TAG56:7-FA20:5	uM or %mole
Primary	Neutral Lipids	TAG	Lipid Species	TAG56:7-FA22:4	uM or %mole
Primary	Neutral Lipids	TAG	Lipid Species	TAG56:7-FA22:5	uM or %mole
Primary	Neutral Lipids	TAG	Lipid Species	TAG56:7-FA22:6	uM or %mole
Primary	Neutral Lipids	TAG	Lipid Species	TAG56:8-FA16:0	uM or %mole
Primary	Neutral Lipids	TAG	Lipid Species	TAG56:8-FA16:1	uM or %mole
Primary	Neutral Lipids	TAG	Lipid Species	TAG56:8-FA18:1	uM or %mole
Primary	Neutral Lipids	TAG	Lipid Species	TAG56:8-FA18:2	uM or %mole
Primary	Neutral Lipids	TAG	Lipid Species	TAG56:8-FA18:3	uM or %mole
Primary	Neutral Lipids	TAG	Lipid Species	TAG56:8-FA20:4	uM or %mole
Primary	Neutral Lipids	TAG	Lipid Species	TAG56:8-FA20:5	uM or %mole

Primary	Neutral Lipids	TAG	Lipid Species	TAG56:8-FA22:5	uM or %mole
Primary	Neutral Lipids	TAG	Lipid Species	TAG56:8-FA22:6	uM or %mole
Primary	Neutral Lipids	TAG	Lipid Species	TAG56:9-FA18:3	uM or %mole
Primary	Neutral Lipids	TAG	Lipid Species	TAG56:9-FA20:4	uM or %mole
Primary	Neutral Lipids	TAG	Lipid Species	TAG56:9-FA20:5	uM or %mole
Primary	Neutral Lipids	TAG	Lipid Species	TAG56:9-FA22:6	uM or %mole
Primary	Neutral Lipids	TAG	Lipid Species	TAG57:2-FA18:1	uM or %mole
Primary	Neutral Lipids	TAG	Lipid Species	TAG57:3-FA18:2	uM or %mole
Primary	Neutral Lipids	TAG	Lipid Species	TAG57:8-FA22:6	uM or %mole
Primary	Neutral Lipids	TAG	Lipid Species	TAG57:9-FA22:6	uM or %mole
Primary	Neutral Lipids	TAG	Lipid Species	TAG58:10-FA18:2	uM or %mole
Primary	Neutral Lipids	TAG	Lipid Species	TAG58:10-FA20:4	uM or %mole
Primary	Neutral Lipids	TAG	Lipid Species	TAG58:10-FA20:5	uM or %mole
Primary	Neutral Lipids	TAG	Lipid Species	TAG58:10-FA22:5	uM or %mole
Primary	Neutral Lipids	TAG	Lipid Species	TAG58:10-FA22:6	uM or %mole
Primary	Neutral Lipids	TAG	Lipid Species	TAG58:2-FA18:1	uM or %mole
Primary	Neutral Lipids	TAG	Lipid Species	TAG58:3-FA18:1	uM or %mole
Primary	Neutral Lipids	TAG	Lipid Species	TAG58:5-FA18:1	uM or %mole
Primary	Neutral Lipids	TAG	Lipid Species	TAG58:6-FA16:0	uM or %mole
Primary	Neutral Lipids	TAG	Lipid Species	TAG58:6-FA18:0	uM or %mole
Primary	Neutral Lipids	TAG	Lipid Species	TAG58:6-FA18:1	uM or %mole
Primary	Neutral Lipids	TAG	Lipid Species	TAG58:6-FA20:4	uM or %mole
Primary	Neutral Lipids	TAG	Lipid Species	TAG58:6-FA22:4	uM or %mole
Primary	Neutral Lipids	TAG	Lipid Species	TAG58:6-FA22:5	uM or %mole
Primary	Neutral Lipids	TAG	Lipid Species	TAG58:7-FA16:0	uM or %mole
Primary	Neutral Lipids	TAG	Lipid Species	TAG58:7-FA18:0	uM or %mole
Primary	Neutral Lipids	TAG	Lipid Species	TAG58:7-FA18:1	uM or %mole
Primary	Neutral Lipids	TAG	Lipid Species	TAG58:7-FA18:2	uM or %mole
Primary	Neutral Lipids	TAG	Lipid Species	TAG58:7-FA20:4	uM or %mole
Primary	Neutral Lipids	TAG	Lipid Species	TAG58:7-FA22:4	uM or %mole
Primary	Neutral Lipids	TAG	Lipid Species	TAG58:7-FA22:5	uM or %mole
Primary	Neutral Lipids	TAG	Lipid Species	TAG58:7-FA22:6	uM or %mole
Primary	Neutral Lipids	TAG	Lipid Species	TAG58:8-FA18:1	uM or %mole
Primary	Neutral Lipids	TAG	Lipid Species	TAG58:8-FA18:2	uM or %mole
Primary	Neutral Lipids	TAG	Lipid Species	TAG58:8-FA20:3	uM or %mole
Primary	Neutral Lipids	TAG	Lipid Species	TAG58:8-FA20:4	uM or %mole
Primary	Neutral Lipids	TAG	Lipid Species	TAG58:8-FA22:5	uM or %mole
Primary	Neutral Lipids	TAG	Lipid Species	TAG58:8-FA22:6	uM or %mole
Primary	Neutral Lipids	TAG	Lipid Species	TAG58:9-FA18:1	uM or %mole
Primary	Neutral Lipids	TAG	Lipid Species	TAG58:9-FA18:2	uM or %mole
Primary	Neutral Lipids	TAG	Lipid Species	TAG58:9-FA20:4	uM or %mole
Primary	Neutral Lipids	TAG	Lipid Species	TAG58:9-FA22:5	uM or %mole
Primary	Neutral Lipids	TAG	Lipid Species	TAG58:9-FA22:6	uM or %mole
Primary	Neutral Lipids	TAG	Lipid Species	TAG60:10-FA22:5	uM or %mole

Primary	Neutral Lipids	TAG	Lipid Species	TAG60:10-FA22:6	uM or %mole
Primary	Neutral Lipids	TAG	Lipid Species	TAG60:11-FA22:5	uM or %mole
Primary	Neutral Lipids	TAG	Lipid Species	TAG60:11-FA22:6	uM or %mole
Primary	Neutral Lipids	TAG	Lipid Species	TAG60:12-FA22:6	uM or %mole
Primary	Phospholipids	LPC	Lipid Species	LPC(14:0)	uM or %mole
Primary	Phospholipids	LPC	Lipid Species	LPC(15:0)	uM or %mole
Primary	Phospholipids	LPC	Lipid Species	LPC(16:0)	uM or %mole
Primary	Phospholipids	LPC	Lipid Species	LPC(16:1)	uM or %mole
Primary	Phospholipids	LPC	Lipid Species	LPC(17:0)	uM or %mole
Primary	Phospholipids	LPC	Lipid Species	LPC(18:0)	uM or %mole
Primary	Phospholipids	LPC	Lipid Species	LPC(18:1)	uM or %mole
Primary	Phospholipids	LPC	Lipid Species	LPC(18:2)	uM or %mole
Primary	Phospholipids	LPC	Lipid Species	LPC(18:3)	uM or %mole
Primary	Phospholipids	LPC	Lipid Species	LPC(20:0)	uM or %mole
Primary	Phospholipids	LPC	Lipid Species	LPC(20:1)	uM or %mole
Primary	Phospholipids	LPC	Lipid Species	LPC(20:2)	uM or %mole
Primary	Phospholipids	LPC	Lipid Species	LPC(20:3)	uM or %mole
Primary	Phospholipids	LPC	Lipid Species	LPC(20:4)	uM or %mole
Primary	Phospholipids	LPC	Lipid Species	LPC(20:5)	uM or %mole
Primary	Phospholipids	LPC	Lipid Species	LPC(22:1)	uM or %mole
Primary	Phospholipids	LPC	Lipid Species	LPC(22:2)	uM or %mole
Primary	Phospholipids	LPC	Lipid Species	LPC(22:4)	uM or %mole
Primary	Phospholipids	LPC	Lipid Species	LPC(22:5)	uM or %mole
Primary	Phospholipids	LPC	Lipid Species	LPC(22:6)	uM or %mole
Primary	Phospholipids	LPE	Lipid Species	LPE(15:0)	uM or %mole
Primary	Phospholipids	LPE	Lipid Species	LPE(16:0)	uM or %mole
Primary	Phospholipids	LPE	Lipid Species	LPE(16:1)	uM or %mole
Primary	Phospholipids	LPE	Lipid Species	LPE(17:0)	uM or %mole
Primary	Phospholipids	LPE	Lipid Species	LPE(18:0)	uM or %mole
Primary	Phospholipids	LPE	Lipid Species	LPE(18:1)	uM or %mole
Primary	Phospholipids	LPE	Lipid Species	LPE(18:2)	uM or %mole
Primary	Phospholipids	LPE	Lipid Species	LPE(18:3)	uM or %mole
Primary	Phospholipids	LPE	Lipid Species	LPE(20:1)	uM or %mole
Primary	Phospholipids	LPE	Lipid Species	LPE(20:2)	uM or %mole
Primary	Phospholipids	LPE	Lipid Species	LPE(20:3)	uM or %mole
Primary	Phospholipids	LPE	Lipid Species	LPE(20:4)	uM or %mole
Primary	Phospholipids	LPE	Lipid Species	LPE(20:5)	uM or %mole
Primary	Phospholipids	LPE	Lipid Species	LPE(22:5)	uM or %mole
Primary	Phospholipids	LPE	Lipid Species	LPE(22:6)	uM or %mole
Primary	Phospholipids	PC	Lipid Species	PC(12:0/18:1)	uM or %mole
Primary	Phospholipids	PC	Lipid Species	PC(12:0/18:2)	uM or %mole
Primary	Phospholipids	PC	Lipid Species	PC(12:0/18:3)	uM or %mole
Primary	Phospholipids	PC	Lipid Species	PC(14:0/16:1)	uM or %mole
Primary	Phospholipids	PC	Lipid Species	PC(14:0/18:1)	uM or %mole
Primary	Phospholipids	PC	Lipid Species	PC(14:0/18:2)	uM or %mole

Primary	Phospholipids	PC	Lipid Species	PC(14:0/18:3)	uM or %mole
Primary	Phospholipids	PC	Lipid Species	PC(14:0/20:2)	uM or %mole
Primary	Phospholipids	PC	Lipid Species	PC(14:0/20:3)	uM or %mole
Primary	Phospholipids	PC	Lipid Species	PC(14:0/20:4)	uM or %mole
Primary	Phospholipids	PC	Lipid Species	PC(15:0/18:1)	uM or %mole
Primary	Phospholipids	PC	Lipid Species	PC(15:0/18:2)	uM or %mole
Primary	Phospholipids	PC	Lipid Species	PC(16:0/12:0)	uM or %mole
Primary	Phospholipids	PC	Lipid Species	PC(16:0/14:0)	uM or %mole
Primary	Phospholipids	PC	Lipid Species	PC(16:0/16:0)	uM or %mole
Primary	Phospholipids	PC	Lipid Species	PC(16:0/16:1)	uM or %mole
Primary	Phospholipids	PC	Lipid Species	PC(16:0/18:0)	uM or %mole
Primary	Phospholipids	PC	Lipid Species	PC(16:0/18:1)	uM or %mole
Primary	Phospholipids	PC	Lipid Species	PC(16:0/18:2)	uM or %mole
Primary	Phospholipids	PC	Lipid Species	PC(16:0/18:3)	uM or %mole
Primary	Phospholipids	PC	Lipid Species	PC(16:0/20:1)	uM or %mole
Primary	Phospholipids	PC	Lipid Species	PC(16:0/20:2)	uM or %mole
Primary	Phospholipids	PC	Lipid Species	PC(16:0/20:3)	uM or %mole
Primary	Phospholipids	PC	Lipid Species	PC(16:0/20:4)	uM or %mole
Primary	Phospholipids	PC	Lipid Species	PC(16:0/22:1)	uM or %mole
Primary	Phospholipids	PC	Lipid Species	PC(16:0/22:2)	uM or %mole
Primary	Phospholipids	PC	Lipid Species	PC(16:0/22:4)	uM or %mole
Primary	Phospholipids	PC	Lipid Species	PC(16:0/22:5)	uM or %mole
Primary	Phospholipids	PC	Lipid Species	PC(16:0/22:6)	uM or %mole
Primary	Phospholipids	PC	Lipid Species	PC(17:0/18:1)	uM or %mole
Primary	Phospholipids	PC	Lipid Species	PC(17:0/18:2)	uM or %mole
Primary	Phospholipids	PC	Lipid Species	PC(17:0/20:3)	uM or %mole
Primary	Phospholipids	PC	Lipid Species	PC(17:0/20:4)	uM or %mole
Primary	Phospholipids	PC	Lipid Species	PC(18:0/12:0)	uM or %mole
Primary	Phospholipids	PC	Lipid Species	PC(18:0/14:0)	uM or %mole
Primary	Phospholipids	PC	Lipid Species	PC(18:0/16:1)	uM or %mole
Primary	Phospholipids	PC	Lipid Species	PC(18:0/18:0)	uM or %mole
Primary	Phospholipids	PC	Lipid Species	PC(18:0/18:1)	uM or %mole
Primary	Phospholipids	PC	Lipid Species	PC(18:0/18:2)	uM or %mole
Primary	Phospholipids	PC	Lipid Species	PC(18:0/18:3)	uM or %mole
Primary	Phospholipids	PC	Lipid Species	PC(18:0/20:0)	uM or %mole
Primary	Phospholipids	PC	Lipid Species	PC(18:0/20:1)	uM or %mole
Primary	Phospholipids	PC	Lipid Species	PC(18:0/20:2)	uM or %mole
Primary	Phospholipids	PC	Lipid Species	PC(18:0/20:3)	uM or %mole
Primary	Phospholipids	PC	Lipid Species	PC(18:0/20:4)	uM or %mole
Primary	Phospholipids	PC	Lipid Species	PC(18:0/22:2)	uM or %mole
Primary	Phospholipids	PC	Lipid Species	PC(18:0/22:4)	uM or %mole
Primary	Phospholipids	PC	Lipid Species	PC(18:0/22:5)	uM or %mole
Primary	Phospholipids	PC	Lipid Species	PC(18:0/22:6)	uM or %mole
Primary	Phospholipids	PC	Lipid Species	PC(18:1/16:1)	uM or %mole

Primary	Phospholipids	PC	Lipid Species	PC(18:1/18:1)	uM or %mole
Primary	Phospholipids	PC	Lipid Species	PC(18:1/18:2)	uM or %mole
Primary	Phospholipids	PC	Lipid Species	PC(18:1/18:3)	uM or %mole
Primary	Phospholipids	PC	Lipid Species	PC(18:1/20:1)	uM or %mole
Primary	Phospholipids	PC	Lipid Species	PC(18:1/20:2)	uM or %mole
Primary	Phospholipids	PC	Lipid Species	PC(18:1/20:3)	uM or %mole
Primary	Phospholipids	PC	Lipid Species	PC(18:1/20:4)	uM or %mole
Primary	Phospholipids	PC	Lipid Species	PC(18:1/22:6)	uM or %mole
Primary	Phospholipids	PC	Lipid Species	PC(18:2/16:1)	uM or %mole
Primary	Phospholipids	PC	Lipid Species	PC(18:2/18:2)	uM or %mole
Primary	Phospholipids	PC	Lipid Species	PC(18:2/18:3)	uM or %mole
Primary	Phospholipids	PC	Lipid Species	PC(18:2/20:1)	uM or %mole
Primary	Phospholipids	PC	Lipid Species	PC(18:2/20:2)	uM or %mole
Primary	Phospholipids	PC	Lipid Species	PC(18:2/20:3)	uM or %mole
Primary	Phospholipids	PC	Lipid Species	PC(18:2/20:4)	uM or %mole
Primary	Phospholipids	PC	Lipid Species	PC(20:0/14:1)	uM or %mole
Primary	Phospholipids	PC	Lipid Species	PC(20:0/18:1)	uM or %mole
Primary	Phospholipids	PC	Lipid Species	PC(20:0/18:2)	uM or %mole
Primary	Phospholipids	PC	Lipid Species	PC(20:0/20:4)	uM or %mole
Primary	Phospholipids	PE	Lipid Species	PE(16:0/16:0)	uM or %mole
Primary	Phospholipids	PE	Lipid Species	PE(16:0/18:1)	uM or %mole
Primary	Phospholipids	PE	Lipid Species	PE(16:0/18:2)	uM or %mole
Primary	Phospholipids	PE	Lipid Species	PE(16:0/20:3)	uM or %mole
Primary	Phospholipids	PE	Lipid Species	PE(16:0/20:4)	uM or %mole
Primary	Phospholipids	PE	Lipid Species	PE(16:0/22:5)	uM or %mole
Primary	Phospholipids	PE	Lipid Species	PE(16:0/22:6)	uM or %mole
Primary	Phospholipids	PE	Lipid Species	PE(18:0/18:0)	uM or %mole
Primary	Phospholipids	PE	Lipid Species	PE(18:0/18:1)	uM or %mole
Primary	Phospholipids	PE	Lipid Species	PE(18:0/18:2)	uM or %mole
Primary	Phospholipids	PE	Lipid Species	PE(18:0/18:3)	uM or %mole
Primary	Phospholipids	PE	Lipid Species	PE(18:0/20:1)	uM or %mole
Primary	Phospholipids	PE	Lipid Species	PE(18:0/20:2)	uM or %mole
Primary	Phospholipids	PE	Lipid Species	PE(18:0/20:3)	uM or %mole
Primary	Phospholipids	PE	Lipid Species	PE(18:0/20:4)	uM or %mole
Primary	Phospholipids	PE	Lipid Species	PE(18:0/20:5)	uM or %mole
Primary	Phospholipids	PE	Lipid Species	PE(18:0/22:2)	uM or %mole
Primary	Phospholipids	PE	Lipid Species	PE(18:0/22:4)	uM or %mole
Primary	Phospholipids	PE	Lipid Species	PE(18:0/22:5)	uM or %mole
Primary	Phospholipids	PE	Lipid Species	PE(18:0/22:6)	uM or %mole
Primary	Phospholipids	PE	Lipid Species	PE(18:1/16:1)	uM or %mole
Primary	Phospholipids	PE	Lipid Species	PE(18:1/18:1)	uM or %mole
Primary	Phospholipids	PE	Lipid Species	PE(18:1/18:2)	uM or %mole
Primary	Phospholipids	PE	Lipid Species	PE(18:1/20:4)	uM or %mole
Primary	Phospholipids	PE	Lipid Species	PE(18:1/22:0)	uM or %mole

Primary	Phospholipids	PE	Lipid Species	PE(18:2/16:1)	uM or %mole
Primary	Phospholipids	PE	Lipid Species	PE(O-16:0/18:1)	uM or %mole
Primary	Phospholipids	PE	Lipid Species	PE(O-16:0/18:2)	uM or %mole
Primary	Phospholipids	PE	Lipid Species	PE(O-16:0/20:4)	uM or %mole
Primary	Phospholipids	PE	Lipid Species	PE(O-16:0/22:5)	uM or %mole
Primary	Phospholipids	PE	Lipid Species	PE(O-16:0/22:6)	uM or %mole
Primary	Phospholipids	PE	Lipid Species	PE(O-18:0/16:0)	uM or %mole
Primary	Phospholipids	PE	Lipid Species	PE(O-18:0/18:1)	uM or %mole
Primary	Phospholipids	PE	Lipid Species	PE(O-18:0/18:2)	uM or %mole
Primary	Phospholipids	PE	Lipid Species	PE(O-18:0/20:3)	uM or %mole
Primary	Phospholipids	PE	Lipid Species	PE(O-18:0/20:4)	uM or %mole
Primary	Phospholipids	PE	Lipid Species	PE(P-16:0/16:0)	uM or %mole
Primary	Phospholipids	PE	Lipid Species	PE(P-16:0/18:1)	uM or %mole
Primary	Phospholipids	PE	Lipid Species	PE(P-16:0/18:2)	uM or %mole
Primary	Phospholipids	PE	Lipid Species	PE(P-16:0/20:3)	uM or %mole
Primary	Phospholipids	PE	Lipid Species	PE(P-16:0/20:4)	uM or %mole
Primary	Phospholipids	PE	Lipid Species	PE(P-16:0/20:5)	uM or %mole
Primary	Phospholipids	PE	Lipid Species	PE(P-16:0/22:4)	uM or %mole
Primary	Phospholipids	PE	Lipid Species	PE(P-16:0/22:5)	uM or %mole
Primary	Phospholipids	PE	Lipid Species	PE(P-16:0/22:6)	uM or %mole
Primary	Phospholipids	PE	Lipid Species	PE(P-18:0/16:0)	uM or %mole
Primary	Phospholipids	PE	Lipid Species	PE(P-18:0/18:1)	uM or %mole
Primary	Phospholipids	PE	Lipid Species	PE(P-18:0/18:2)	uM or %mole
Primary	Phospholipids	PE	Lipid Species	PE(P-18:0/20:3)	uM or %mole
Primary	Phospholipids	PE	Lipid Species	PE(P-18:0/20:4)	uM or %mole
Primary	Phospholipids	PE	Lipid Species	PE(P-18:0/20:5)	uM or %mole
Primary	Phospholipids	PE	Lipid Species	PE(P-18:0/22:5)	uM or %mole
Primary	Phospholipids	PE	Lipid Species	PE(P-18:0/22:6)	uM or %mole
Primary	Phospholipids	PE	Lipid Species	PE(P-18:1/16:0)	uM or %mole
Primary	Phospholipids	PE	Lipid Species	PE(P-18:1/18:1)	uM or %mole
Primary	Phospholipids	PE	Lipid Species	PE(P-18:1/18:2)	uM or %mole
Primary	Phospholipids	PE	Lipid Species	PE(P-18:1/20:3)	uM or %mole
Primary	Phospholipids	PE	Lipid Species	PE(P-18:1/20:4)	uM or %mole
Primary	Phospholipids	PE	Lipid Species	PE(P-18:1/20:5)	uM or %mole
Primary	Phospholipids	PE	Lipid Species	PE(P-18:1/22:6)	uM or %mole
Primary	Phospholipids	PI	Lipid Species	PI(16:0/16:0)	uM or %mole
Primary	Phospholipids	PI	Lipid Species	PI(16:0/16:1)	uM or %mole
Primary	Phospholipids	PI	Lipid Species	PI(16:0/18:1)	uM or %mole
Primary	Phospholipids	PI	Lipid Species	PI(16:0/18:2)	uM or %mole
Primary	Phospholipids	PI	Lipid Species	PI(16:0/20:3)	uM or %mole
Primary	Phospholipids	PI	Lipid Species	PI(16:0/20:4)	uM or %mole
Primary	Phospholipids	PI	Lipid Species	PI(18:0/16:0)	uM or %mole
Primary	Phospholipids	PI	Lipid Species	PI(18:0/16:1)	uM or %mole
Primary	Phospholipids	PI	Lipid Species	PI(18:0/18:1)	uM or %mole

Primary	Phospholipids	PI	Lipid Species	PI(18:0/18:2)	uM or %mole
Primary	Phospholipids	PI	Lipid Species	PI(18:0/18:3)	uM or %mole
Primary	Phospholipids	PI	Lipid Species	PI(18:0/20:2)	uM or %mole
Primary	Phospholipids	PI	Lipid Species	PI(18:0/20:3)	uM or %mole
Primary	Phospholipids	PI	Lipid Species	PI(18:0/20:4)	uM or %mole
Primary	Phospholipids	PI	Lipid Species	PI(18:0/22:5)	uM or %mole
Primary	Phospholipids	PI	Lipid Species	PI(18:1/16:1)	uM or %mole
Primary	Phospholipids	PI	Lipid Species	PI(18:1/18:1)	uM or %mole
Primary	Phospholipids	PI	Lipid Species	PI(18:1/18:2)	uM or %mole
Primary	Phospholipids	PI	Lipid Species	PI(18:1/20:4)	uM or %mole
Primary	Sphingolipids	CER	Lipid Species	CER(14:0)	uM or %mole
Primary	Sphingolipids	CER	Lipid Species	CER(16:0)	uM or %mole
Primary	Sphingolipids	CER	Lipid Species	CER(18:0)	uM or %mole
Primary	Sphingolipids	CER	Lipid Species	CER(18:1)	uM or %mole
Primary	Sphingolipids	CER	Lipid Species	CER(20:0)	uM or %mole
Primary	Sphingolipids	CER	Lipid Species	CER(22:0)	uM or %mole
Primary	Sphingolipids	CER	Lipid Species	CER(22:1)	uM or %mole
Primary	Sphingolipids	CER	Lipid Species	CER(24:0)	uM or %mole
Primary	Sphingolipids	CER	Lipid Species	CER(24:1)	uM or %mole
Primary	Sphingolipids	CER	Lipid Species	CER(26:0)	uM or %mole
Primary	Sphingolipids	CER	Lipid Species	CER(26:1)	uM or %mole
Primary	Sphingolipids	DCER	Lipid Species	DCER(16:0)	uM or %mole
Primary	Sphingolipids	DCER	Lipid Species	DCER(18:0)	uM or %mole
Primary	Sphingolipids	DCER	Lipid Species	DCER(20:0)	uM or %mole
Primary	Sphingolipids	DCER	Lipid Species	DCER(22:0)	uM or %mole
Primary	Sphingolipids	DCER	Lipid Species	DCER(24:0)	uM or %mole
Primary	Sphingolipids	DCER	Lipid Species	DCER(24:1)	uM or %mole
Primary	Sphingolipids	DCER	Lipid Species	DCER(26:0)	uM or %mole
Primary	Sphingolipids	HCER	Lipid Species	HCER(16:0)	uM or %mole
Primary	Sphingolipids	HCER	Lipid Species	HCER(18:0)	uM or %mole
Primary	Sphingolipids	HCER	Lipid Species	HCER(18:1)	uM or %mole
Primary	Sphingolipids	HCER	Lipid Species	HCER(20:0)	uM or %mole
Primary	Sphingolipids	HCER	Lipid Species	HCER(20:1)	uM or %mole
Primary	Sphingolipids	HCER	Lipid Species	HCER(22:0)	uM or %mole
Primary	Sphingolipids	HCER	Lipid Species	HCER(22:1)	uM or %mole
Primary	Sphingolipids	HCER	Lipid Species	HCER(24:0)	uM or %mole
Primary	Sphingolipids	HCER	Lipid Species	HCER(24:1)	uM or %mole
Primary	Sphingolipids	HCER	Lipid Species	HCER(26:0)	uM or %mole
Primary	Sphingolipids	HCER	Lipid Species	HCER(26:1)	uM or %mole
Primary	Sphingolipids	LCER	Lipid Species	LCER(14:0)	uM or %mole
Primary	Sphingolipids	LCER	Lipid Species	LCER(16:0)	uM or %mole
Primary	Sphingolipids	LCER	Lipid Species	LCER(18:0)	uM or %mole
Primary	Sphingolipids	LCER	Lipid Species	LCER(18:1)	uM or %mole
Primary	Sphingolipids	LCER	Lipid Species	LCER(20:0)	uM or %mole

Primary	Sphingolipids	LCER	Lipid Species	LCER(20:1)	uM or %mole
Primary	Sphingolipids	LCER	Lipid Species	LCER(22:0)	uM or %mole
Primary	Sphingolipids	LCER	Lipid Species	LCER(22:1)	uM or %mole
Primary	Sphingolipids	LCER	Lipid Species	LCER(24:0)	uM or %mole
Primary	Sphingolipids	LCER	Lipid Species	LCER(24:1)	uM or %mole
Primary	Sphingolipids	LCER	Lipid Species	LCER(26:0)	uM or %mole
Primary	Sphingolipids	LCER	Lipid Species	LCER(26:1)	uM or %mole
Primary	Sphingolipids	SM	Lipid Species	SM(14:0)	uM or %mole
Primary	Sphingolipids	SM	Lipid Species	SM(16:0)	uM or %mole
Primary	Sphingolipids	SM	Lipid Species	SM(18:0)	uM or %mole
Primary	Sphingolipids	SM	Lipid Species	SM(18:1)	uM or %mole
Primary	Sphingolipids	SM	Lipid Species	SM(20:0)	uM or %mole
Primary	Sphingolipids	SM	Lipid Species	SM(20:1)	uM or %mole
Primary	Sphingolipids	SM	Lipid Species	SM(22:0)	uM or %mole
Primary	Sphingolipids	SM	Lipid Species	SM(22:1)	uM or %mole
Primary	Sphingolipids	SM	Lipid Species	SM(24:0)	uM or %mole
Primary	Sphingolipids	SM	Lipid Species	SM(24:1)	uM or %mole
Primary	Sphingolipids	SM	Lipid Species	SM(26:0)	uM or %mole
Primary	Sphingolipids	SM	Lipid Species	SM(26:1)	uM or %mole
Secondary	Neutral Lipids	DAG	Fatty acid	DAG[FA12:0]	uM
Secondary	Neutral Lipids	DAG	Fatty acid	DAG[FA14:0]	uM
Secondary	Neutral Lipids	DAG	Fatty acid	DAG[FA14:1]	uM
Secondary	Neutral Lipids	DAG	Fatty acid	DAG[FA15:0]	uM
Secondary	Neutral Lipids	DAG	Fatty acid	DAG[FA16:0]	uM
Secondary	Neutral Lipids	DAG	Fatty acid	DAG[FA16:1]	uM
Secondary	Neutral Lipids	DAG	Fatty acid	DAG[FA18:0]	uM
Secondary	Neutral Lipids	DAG	Fatty acid	DAG[FA18:1]	uM
Secondary	Neutral Lipids	DAG	Fatty acid	DAG[FA18:2]	uM
Secondary	Neutral Lipids	DAG	Fatty acid	DAG[FA18:3]	uM
Secondary	Neutral Lipids	DAG	Fatty acid	DAG[FA20:0]	uM
Secondary	Neutral Lipids	DAG	Fatty acid	DAG[FA20:1]	uM
Secondary	Neutral Lipids	DAG	Fatty acid	DAG[FA20:2]	uM
Secondary	Neutral Lipids	DAG	Fatty acid	DAG[FA20:3]	uM
Secondary	Neutral Lipids	DAG	Fatty acid	DAG[FA20:4]	uM
Secondary	Neutral Lipids	DAG	Fatty acid	DAG[FA22:4]	uM
Secondary	Neutral Lipids	DAG	Fatty acid	DAG[FA22:6]	uM
Secondary	Neutral Lipids	TAG	Fatty acid	TAG[FA12:0]	uM
Secondary	Neutral Lipids	TAG	Fatty acid	TAG[FA14:0]	uM
Secondary	Neutral Lipids	TAG	Fatty acid	TAG[FA14:1]	uM
Secondary	Neutral Lipids	TAG	Fatty acid	TAG[FA15:0]	uM
Secondary	Neutral Lipids	TAG	Fatty acid	TAG[FA16:0]	uM
Secondary	Neutral Lipids	TAG	Fatty acid	TAG[FA16:1]	uM
Secondary	Neutral Lipids	TAG	Fatty acid	TAG[FA17:0]	uM
Secondary	Neutral Lipids	TAG	Fatty acid	TAG[FA18:0]	uM

Secondary	Neutral Lipids	TAG	Fatty acid	TAG[FA18:1]	uM
Secondary	Neutral Lipids	TAG	Fatty acid	TAG[FA18:2]	uM
Secondary	Neutral Lipids	TAG	Fatty acid	TAG[FA18:3]	uM
Secondary	Neutral Lipids	TAG	Fatty acid	TAG[FA20:0]	uM
Secondary	Neutral Lipids	TAG	Fatty acid	TAG[FA20:1]	uM
Secondary	Neutral Lipids	TAG	Fatty acid	TAG[FA20:2]	uM
Secondary	Neutral Lipids	TAG	Fatty acid	TAG[FA20:3]	uM
Secondary	Neutral Lipids	TAG	Fatty acid	TAG[FA20:4]	uM
Secondary	Neutral Lipids	TAG	Fatty acid	TAG[FA20:5]	uM
Secondary	Neutral Lipids	TAG	Fatty acid	TAG[FA22:1]	uM
Secondary	Neutral Lipids	TAG	Fatty acid	TAG[FA22:4]	uM
Secondary	Neutral Lipids	TAG	Fatty acid	TAG[FA22:5]	uM
Secondary	Neutral Lipids	TAG	Fatty acid	TAG[FA22:6]	uM
Secondary	Phospholipids	PC	Fatty acid	PC[FA12:0]	uM
Secondary	Phospholipids	PC	Fatty acid	PC[FA14:0]	uM
Secondary	Phospholipids	PC	Fatty acid	PC[FA14:1]	uM
Secondary	Phospholipids	PC	Fatty acid	PC[FA15:0]	uM
Secondary	Phospholipids	PC	Fatty acid	PC[FA16:0]	uM
Secondary	Phospholipids	PC	Fatty acid	PC[FA16:1]	uM
Secondary	Phospholipids	PC	Fatty acid	PC[FA17:0]	uM
Secondary	Phospholipids	PC	Fatty acid	PC[FA18:0]	uM
Secondary	Phospholipids	PC	Fatty acid	PC[FA18:1]	uM
Secondary	Phospholipids	PC	Fatty acid	PC[FA18:2]	uM
Secondary	Phospholipids	PC	Fatty acid	PC[FA18:3]	uM
Secondary	Phospholipids	PC	Fatty acid	PC[FA20:0]	uM
Secondary	Phospholipids	PC	Fatty acid	PC[FA20:1]	uM
Secondary	Phospholipids	PC	Fatty acid	PC[FA20:2]	uM
Secondary	Phospholipids	PC	Fatty acid	PC[FA20:3]	uM
Secondary	Phospholipids	PC	Fatty acid	PC[FA20:4]	uM
Secondary	Phospholipids	PC	Fatty acid	PC[FA22:1]	uM
Secondary	Phospholipids	PC	Fatty acid	PC[FA22:2]	uM
Secondary	Phospholipids	PC	Fatty acid	PC[FA22:4]	uM
Secondary	Phospholipids	PC	Fatty acid	PC[FA22:5]	uM
Secondary	Phospholipids	PC	Fatty acid	PC[FA22:6]	uM
Secondary	Phospholipids	PE	Fatty acid	PE[FA16:0]	uM
Secondary	Phospholipids	PE	Fatty acid	PE[FA16:1]	uM
Secondary	Phospholipids	PE	Fatty acid	PE[FA18:0]	uM
Secondary	Phospholipids	PE	Fatty acid	PE[FA18:1]	uM
Secondary	Phospholipids	PE	Fatty acid	PE[FA18:2]	uM
Secondary	Phospholipids	PE	Fatty acid	PE[FA18:3]	uM
Secondary	Phospholipids	PE	Fatty acid	PE[FA20:1]	uM
Secondary	Phospholipids	PE	Fatty acid	PE[FA20:2]	uM
Secondary	Phospholipids	PE	Fatty acid	PE[FA20:3]	uM
Secondary	Phospholipids	PE	Fatty acid	PE[FA20:4]	uM

Secondary	Phospholipids	PE	Fatty acid	PE[FA20:5]	uM
Secondary	Phospholipids	PE	Fatty acid	PE[FA22:0]	uM
Secondary	Phospholipids	PE	Fatty acid	PE[FA22:2]	uM
Secondary	Phospholipids	PE	Fatty acid	PE[FA22:4]	uM
Secondary	Phospholipids	PE	Fatty acid	PE[FA22:5]	uM
Secondary	Phospholipids	PE	Fatty acid	PE[FA22:6]	uM
Secondary	Phospholipids	PI	Fatty acid	PI[FA16:0]	uM
Secondary	Phospholipids	PI	Fatty acid	PI[FA16:1]	uM
Secondary	Phospholipids	PI	Fatty acid	PI[FA18:0]	uM
Secondary	Phospholipids	PI	Fatty acid	PI[FA18:1]	uM
Secondary	Phospholipids	PI	Fatty acid	PI[FA18:2]	uM
Secondary	Phospholipids	PI	Fatty acid	PI[FA18:3]	uM
Secondary	Phospholipids	PI	Fatty acid	PI[FA20:2]	uM
Secondary	Phospholipids	PI	Fatty acid	PI[FA20:3]	uM
Secondary	Phospholipids	PI	Fatty acid	PI[FA20:4]	uM
Secondary	Phospholipids	PI	Fatty acid	PI[FA22:5]	uM
Secondary	Neutral Lipids	CE	Lipid class	CE	uM
Secondary	Neutral Lipids	TAG	Lipid class	TAG	uM
Secondary	Neutral Lipids	DAG	Lipid class	DAG	uM
Secondary	Neutral Lipids	FFA	Lipid class	FFA	uM
Secondary	Phospholipids	PC	Lipid class	PC	uM
Secondary	Phospholipids	PE	Lipid class	PE	uM
Secondary	Phospholipids	PI	Lipid class	PI	uM
Secondary	Phospholipids	LPC	Lipid class	LPC	uM
Secondary	Phospholipids	LPE	Lipid class	LPE	uM
Secondary	Sphingolipids	SM	Lipid class	SM	uM
Secondary	Sphingolipids	CER	Lipid class	CER	uM
Secondary	Sphingolipids	HCER	Lipid class	HCER	uM
Secondary	Sphingolipids	LCER	Lipid class	LCER	uM
Secondary	Sphingolipids	DCER	Lipid class	DCER	uM

Table S4: Metabolite markers of coffee response reported by Cornelis et al, 2016*

Super Pathway	Sub Pathway‡	Metabolite
Amino Acid	Creatine Metabolism	creatinine
		guanidinoacetate
	Histidine Metabolism	hydantoin-5-propionic acid
		imidazole lactate
	Leucine, Isoleucine and Valine Metabolism	isovalerylcarnitine
	Methionine, Cysteine, SAM and Taurine Metabolism	cysteine
		methionine sulfone
	Polyamine Metabolism	4-acetamidobutanoate
		N-acetylputrescine
	Tryptophan Metabolism	5-bromotryptophan
		indolelactate
		kynurenine
	Tyrosine Metabolism	2-hydroxyphenylacetate
	Urea cycle; Arginine and Proline Metabolism	homoarginine
Carbohydrate	Aminosugar Metabolism	glucuronate
	Glycolysis, Gluconeogenesis, and Pyruvate	1,5-anhydroglucitol (1,5-AG)
Cofactors & Vitamins	Nicotinate and Nicotinamide Metabolism	trigonelline (N'-methylnicotinate)
Energy	Oxidative Phosphorylation	phosphate
	TCA Cycle	citraconate/glutaconate
Lipid	Diacylglycerol	linoleoyl-linoleoyl-glycerol (18:2/18:2)[1]*
	Endocannabinoid	linoleoyl ethanolamide
		N-oleoyltaurine
		palmitoyl ethanolamide
		stearoyl ethanolamide
	Fatty Acid Metabolism (Acyl Choline)	arachidonoylcholine
		dihomo-linolenoyl-choline
		docosahexaenoylcholine
		oleoylcholine
		palmitoloelycholine
		palmitoylcholine
	Glycerolipid Metabolism	glycerol 3-phosphate
	Phospholipid Metabolism	choline
	Polyunsaturated Fatty Acid (n3 and n6)	arachidonate (20:4n6)
		docosapentaenoate (n6 DPA;
	Secondary Bile Acid Metabolism	glycocholenate sulfate*
	Sphingolipid Metabolism	palmitoyl dihydrosphingomyelin
	Steroid	4-androsten-3alpha,17alpha-diol monosulfate (3)
		4-androsten-3beta,17beta-diol monosulfate (2)
		epiandrosterone sulfate
		etiocholanolone glucuronide
		pregn steroid monosulfate*
	Sterol	3beta,7alpha-dihydroxy-5-
		campesterol
Nucleotide	Purine Metabolism, (Hypo)Xanthine/Inosine	urate
	Purine Metabolism, Adenine containing	N6-carbamoylthreonyladenosine
	Purine Metabolism, Guanine containing	7-methylguanine
	Pyrimidine Metabolism, Uracil containing	2'-deoxyuridine
Peptide	Dipeptide Derivative	N-acetylcarnosine
	Fibrinogen Cleavage Peptide	DSGEGDFXAEGGGVR*
Xenobiotics	Benzoate Metabolism	3-(3-hydroxyphenyl)propionate
		3-(3-hydroxyphenyl)propionate sulfate
		3-hydroxyhippurate
		3-methyl catechol sulfate (1)
		3-phenylpropionate
		4-vinylphenol sulfate
		catechol sulfate
		hippurate

		O-methylcatechol sulfate
	Chemical	3-hydroxypyridine sulfate
		N-methylpipecolate
		succinimide
	Food Component/Plant	cinnamoylglycine
		dihydroferulic acid
		homostachydrine*
		N-(2-furoyl)glycine
		pyrraline
		quinate
	Xanthine Metabolism	1,3,7-trimethylurate
		1,3-dimethylurate
		1,7-dimethylurate
		1-methylurate
		1-methylxanthine
		3,7-dimethylurate
		3-methylxanthine
		5-acetylamino-6-amino-3-methyluracil
		7-methylxanthine
		caffeic acid sulfate
		caffeine
		paraxanthine
		theobromine
		theophylline

*Listed are 82 metabolites of known identity associated with coffee response from our previous metabolomics analysis of these coffee trial samples{Cornelis, 2018 #8582}. These metabolites were measured by UPLC-ESI-MS/MS (nontargeted) and processed as previously described{Cornelis, 2018 #8582}. Mass spectral peaks, retention times, and m/z were used to determine the relative quantities of each metabolite. Twenty-four of these 82 were also lipids but are herein referred to as ‘metabolites’ to distinguish them from the new lipid species data.

‡Colors correspond to color scheme used in **Figure S3**.

Table S5. Population-based lipidomic studies of habitual coffee consumption*

Ref	Study sample	Coffee measurement†	Metabolite measurement‡	Significant LIPID findings
[5]	Germany, EU§<br>N=284, M<br>age: 55-79 y	FFQ<br>2004-2005<br>cups/d	Overnight fasting serum, morning draws, 2006<br>ESI-MS/MS (Biocrates): 363 metabolites	Coffee intake was positively associated with SM species:<br>SM (OH,COOH) x:y — 20:2, 16:2, 18:2, 24:0, 18:1<br>SM (OH) x:y — 20:3, 22:1, 28:0<br>Coffee intake was negatively associated with long- and medium-chain acylcarnitines: C16:1, C10:1, C12:1, C14:1, C6.
[6]	UK, EU<br>N=1003, F, twins<br>age: 58.5±10.45 y	FFQ<br>categories (0 to 6+ cups/d)	Overnight fasting serum<br>Absolute-IDQ Kit p150 (Biocrates): 126 metabolites	Coffee intake was negatively associated with acylcarnitine C10:1.
[7]	Germany, EU<br>N=2380, M/F<br>age: 49.8± 8.9 y	FFQ<br>1994-1998<br>categories (0 to 5+ cups/d)	Serum, fasting status unknown, 1994-1998<br>Absolute-IDQ Kit p150 (Biocrates): 127 metabolites	Diet pattern with high intake of margarine, non-whole-grain bread, meat, and coffee and low intake of butter, pasta/rice, and tea was positively associated with LPCs: C20:4, C18:2.<br>Diet pattern with high intake of butter, garlic, and coffee and low intake of margarine, fresh fruit, and soup was positively associated with SMs, notably OH-C16:1, OH-C14:1, OH-C24:1, OH-C22:2, OH-C22:1
[8]	Germany, EU<br>N=2380, M/F<br>age: 49.8± 8.9 y	FFQ<br>1994-1998<br>categories (0 to 5+ cups/d)	Serum, fasting status unknown, 1994-1998<br>Absolute-IDQTM Kit p150 (Biocrates): 127 metabolites	Limited species details.<br>Acylcarnitines were inversely associated with coffee<br>Sphingomyelins were particularly positively related to coffee.<br>Acyl-alkyl-phosphatidylcholines were positively linked to coffee.<br>Most lysophosphatidylcholines were positively associated with coffee.<br>Diacylphosphatidylcholines were inversely with coffee and cake and cookies.
[9]	Germany, EU<br>N=1610, M/F<br>age: 35-64 y	FFQ<br>1994-1998<br>categories (0 to 5+ cups/d)	Serum, fasting status unknown, 1994-1998<br>Absolute-IDQTM Kit p150 (Biocrates)<br>Hypothesis testing:<br>13 metabolites previously associated with type 2 diabetes<br>Exploratory:<br>113 metabolites	Hypothesis testing:<br>Coffee was inversely associated with diacylphosphatidylcholine C32:1 in M/F.<br>Coffee was positively associated with acyl-alkyl-phosphatidylcholines C34:3, C40:6, and C42:5 in F.<br>Exploratory: none significantly associated with coffee.
[10]	USA, AA<br>Discovery:<br>N=1500, M/F<br>age: 52.9 ± 5.8 y<br>Replication:<br>N=477, M/F<br>age: 52.7 ± 5.7 y	FFQ<br>1987-1989<br>categories (0 to 6+ cups/d)	8-hr + fasting serum, 1987-1989<br>GC/LC-MS (Metabolon): 356 metabolites	No known lipids
[11]	USA, EU<br>N=502, M/F<br>(cancer cases and matched controls)<br>age: 64 ± 5 y	FFQ<br>1993-2001<br>cups/d	Serum, fasting status unknown<br>GC/LC-MS (Metabolon): 412 "knowns" and 231 "unknowns" detected	No known lipids
[12]	USA, EU<br>N=253, M/F<br>(cancer cases, matched controls)<br>Age: 57 ± 9 y	FFQ<br>Total coffee<br>Regular coffee<br>Decaf coffee<br>g/d<br>+55 other diet traits	12-h overnight non-fasting urine sample<br>Nonfasting serum<br>GC/LC-MS (Metabolon): 824 in urine, 648 in serum	No known lipids
[13]	USA, Mix<br>N=1369,<br>nonsmoking PM F<br>Age=68.3± 5.7 y	FFQ<br>1999-2000<br>Total coffee<br>Regular coffee<br>Decaf coffee<br>+90 diet traits	Nonfasting serum (1998-2001)<br>GC/LC-MS (Metabolon): 1186 metabolites	Total coffee: No known lipids<br>Regular coffee: No known lipids<br>Decaf: No known lipids
[14]	Brazil,	2 24-h recalls<br>FFQ	Fasting-plasma<br>FIA-MS/MS and HPLC-MS/MS:	Coffee decreased LPC a C16:1, C18:1, C20:4<br>Coffee increased LPC a C16:0/C16:1, C18:0/18:1 (ratios)

	N=169 (survey 2008-2009), M/F<br>Age: 50.7 ±18.9	Non (0 ml/d), low (≤100 ml/d and high (>100 ml/d) coffee consumers<br>Regular filtered coffee (no one consumed instant, espresso or other brewing methods, no decaf)<br>+ polyphenol intake	Absolute-IDQ p180 Kit (Biocrates)<br>Only 14 LPC species used in analysis	

*Studies examined predominately regular or total (regular and decaffeinated) coffee. Presented studies are those employing targeted or untargeted analysis of lipid classes captured by the current study.

†Includes method of dietary assessment, year of collection, and type of data collected (if reported).

‡Includes fasting status at time of specimen collection, year of blood/urine collection, and platform used for metabolomic profiling (if reported).

§Population ancestry: EU European, AA African American; Sex: M male, F female

Shown are studies applying metabolomic platforms with *potential* for lipid detection.

Annotation for PCs: “aa” indicates that both moieties at the sn-1 and sn-2 position are fatty acids and bound to the glycerol backbone via ester bonds. “ae” denotes that one of the moieties, either in the sn-1 or at sn-2 position is a fatty alcohol and bound via an ether bond. Total number of carbon atoms and double bonds present in both lipid fatty acid chains are denoted as “C x:y”, where x is the total carbon number of both chains and y is the total number of double bonds.

Annotation for SMs: Total number of carbon atoms, the number of double bonds or the presence of hydroxyl group (OH) are indicated only for the
fatty acid in the amide bond under assumption that the backbone is formed by sphingosine (d18:1). Total number of carbon atoms and double bonds present in fatty acid chain is denoted as “C x:y”, where x is the carbon number and y is the number for double bonds

Table S6. Circulating lysophosphatidylcholines and coffee-implicated disease or conditions*

LPC	Increased Risk / Positive Association	Decreased Risk / Negative Association	Null Results
LPC(15:0)		Obesity/BMI [15,16]<br>CRP [16]<br>HCC [17]<br>CVD [18]<br>T2D & related traits [19]	Breat Ca [20]<br>Prostate Ca [20]<br>CRC [20]<br>T2D & related traits [21,22]<br>CVD[23,24]<br>Obesity/BMI [22]
LPC(17:0)	HCC [25]	HCC [17]<br>Obesity/BMI [26]<br>T2D & related traits [27-29]	T2D & related traits [19,21,22]<br>CVD [23,24]<br>Obesity/BMI [22,30]
LPC(18:1)	AD/Cognition[31,32]<br>Aging[31]<br>LDL[33]<br>HDL [33]<br>Lung Ca [34]<br>Obesity/BMI[35]<br>Weight loss[36]<br>Ovarian Ca[37]	Obesity/BMI [15,16,26,30,33,38-40]<br>MS [41]<br>Lung Ca [42]<br>Weight loss [16]<br>CRP [16,38]<br>HCC [17]<br>Chronic renal failure [43]<br>Ovarian Ca [44]<br>CVD [45]<br>CRC [46]<br>T2D & related traits [19,27,47]	Obesity/BMI [22,39,48]<br>T2D & related traits [21,27,28,48]<br>HTN/BP [48]<br>Breast Ca [20]<br>Prostate Ca [20]<br>CRC [20]<br>CVD [23] [24]<br>Mortality [49]<br>Ovarian Ca [50]
LPC(20:2)		T2D & related traits [19]	LDL [33]<br>HDL [33]<br>Obesity/BMI [33,40]<br>CVD [23]
LPC(20:3)	Lung Ca [34]<br>Obesity/BMI [35]<br>Ovarian Ca [37]	Huntington's [51]<br>Weight loss <100 [16]<br>HCC 200 [17] CVD [18]	Breat Ca [20]<br>Prostate Ca [20]<br>CRC [20]<br>CVD [23,24]<br>Obesity/BMI [30,39]<br>T2D & related traits [27,28]
LPC(20:4)	HDL [52]<br>Lung Ca [34]<br>Obesity/BMI [35]<br>Weight loss [36]<br>Ovarian Ca [37]	CVD [52]<br>HTN/BP [52]<br>Obesity/BMI [15,16,30,52]<br>CRP [16]<br>HCC [17]	LDL [33,35]<br>TG [35]<br>HDL [33] [35]<br>Obesity/BMI [22,33,39]<br>Breat Ca [20]<br>Prostate Ca [20]<br>T2D & related traits [19,21,22,27,28]<br>Ovarian Ca [44,50]<br>CVD [23,24]<br>Mortality [49]<br>CRC [20,46]
LPC(22:1)	T2D & related traits [22]		Obesity/BMI [22]<br>T2D & related traits [19,21]
LPC(22:2)			
Total (LPC)	AD/Cognition [31]<br>Aging [31]<br>Lung Ca [34]<br>Depression [53]<br>Ovarian Ca [44]	Obesity/BMI [15,16]<br>Weight loss/RYGB [16,54]<br>CRP [16]<br>Lung function [55]<br>T2D & related traits [21]<br>CVD [45]<br>CRC [46]	AD/Cognition [56]<br>Obesity/BMI [48]<br>T2D & related traits [48]<br>HTN/BP[48]<br>CVD [24]<br>Ovarian Ca [50]

*Shown are results from a non-comprehensive literature search for human clinical or observational studies relating circulating lysophosphatidylcholine with coffee-implicated disease or conditions [57].

Figure S1.

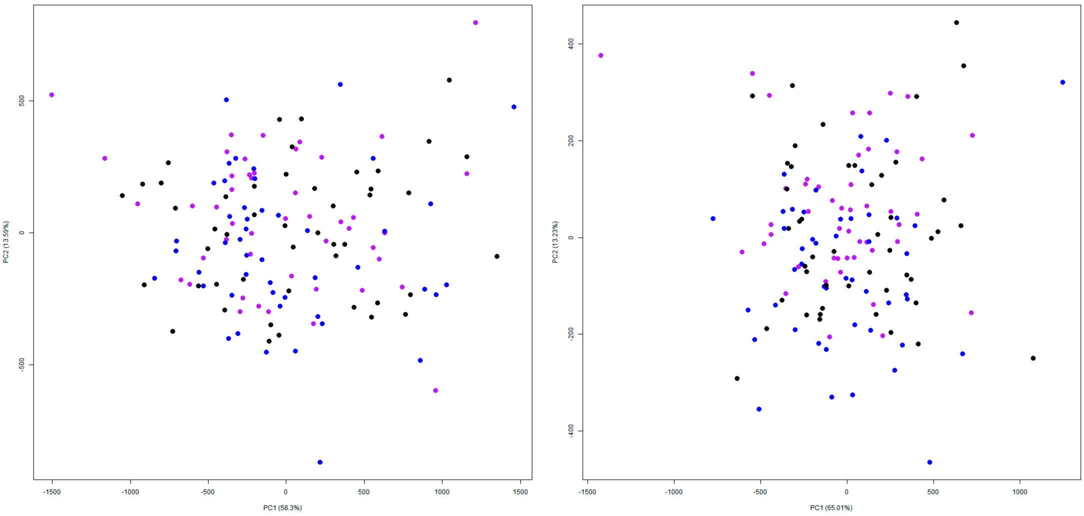

Results from PCA (A) and multilevel PCA (B) analysis. Score plot of the first and second PC. Black, purple and blue points represent samples measured after the 0 cups/d, 4 cups/d and 8 cups/d trial periods, respectively.

Figure S2

A

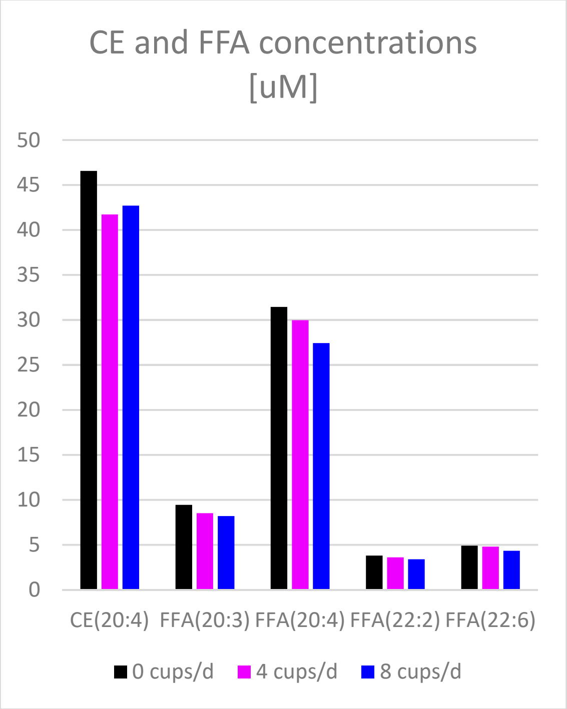

B

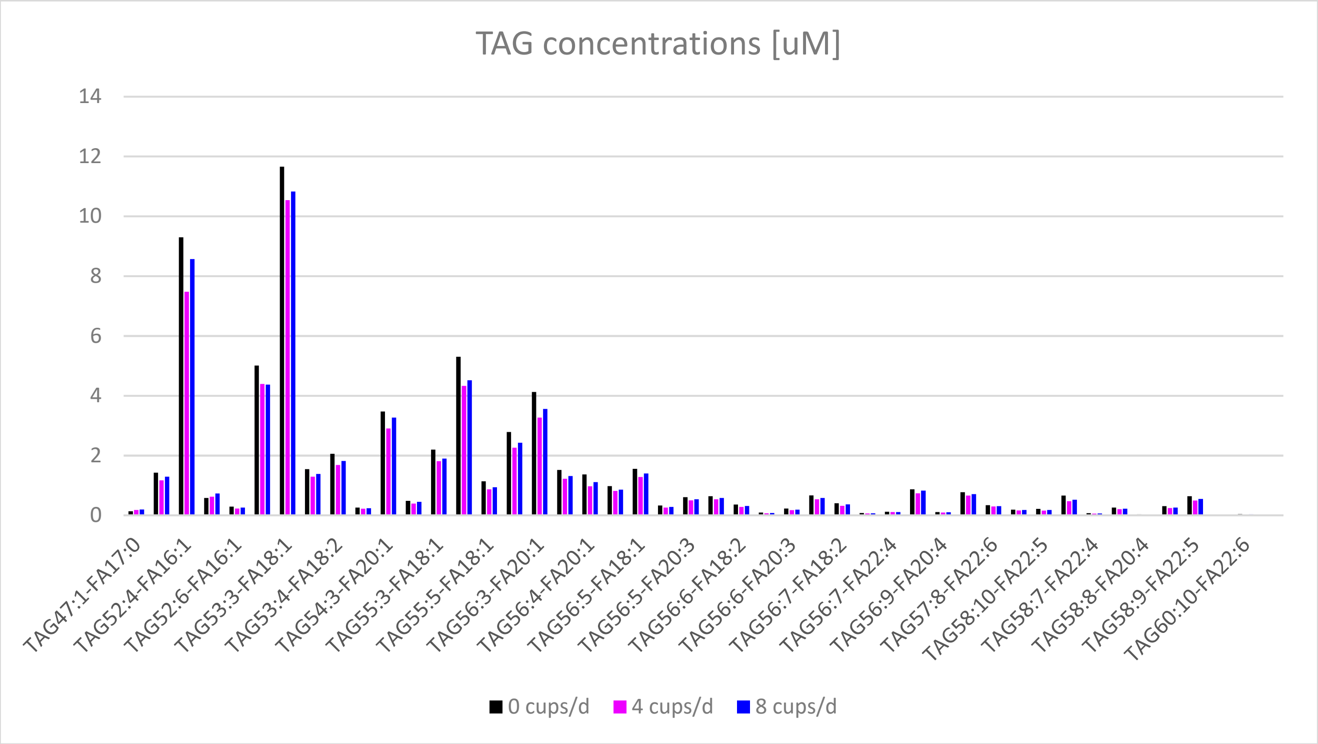

C

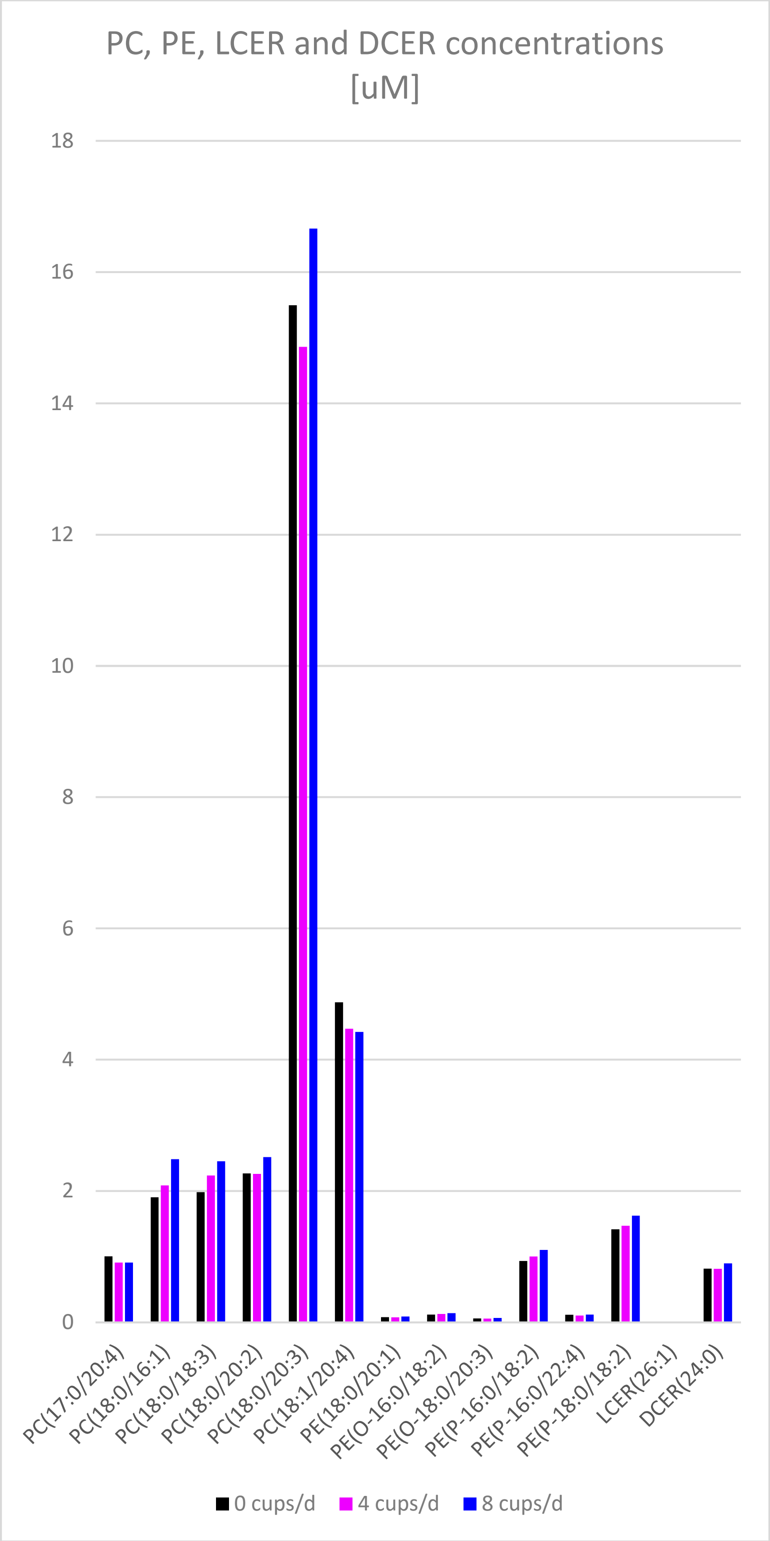

Serum concentrations [uM] of lipid species nominally associated with changes in response to coffee intake (P<0.05, FDR>0.05). Data for TAG54:3-FA18:1 not displayed (0 cups/d: 104.9 uM, 4 cups/d: 86.4 uM, 8 cups/d: 94.3 uM).

Figure S3.

Pearson correlations (r) of changes in variable (lipid/metabolite/biomarker) levels after A) 4 cups/d compared to 0 cups/d B) 8 cups/d compared to 0 cups/d and C) 8 cups/d compared to 4 cups/d. Edges correspond to r and are shown if > |0.50| (corresponding to a nominal P<3.5×10^{-4}). Distances between nodes reflect strength of correlations. Metabolites that did no correlate with lipid species or clinical markers but correlated with other metabolites have been trimmed from the network since these were previously reported in Cornelis et al 2018.

A

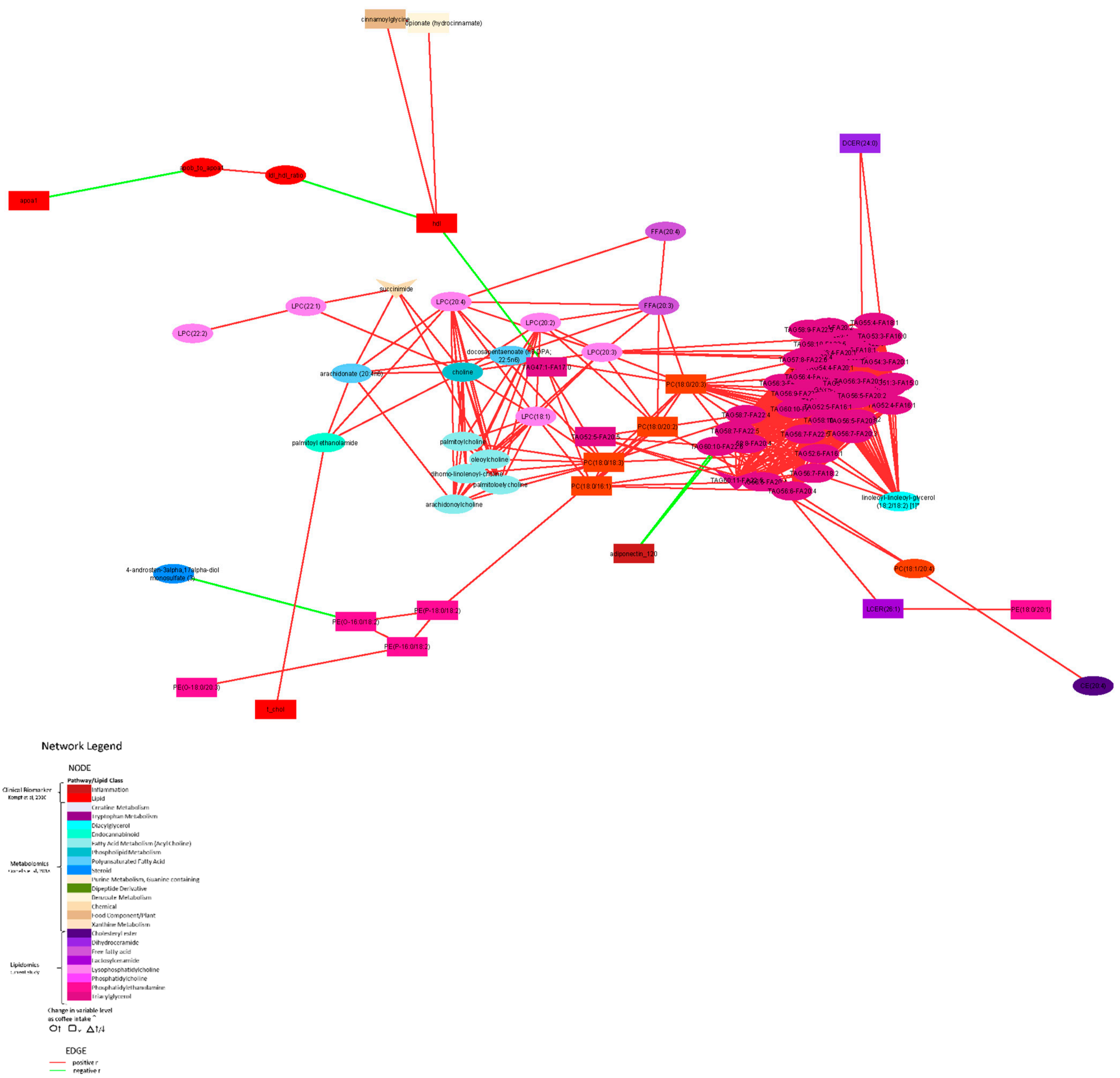

B

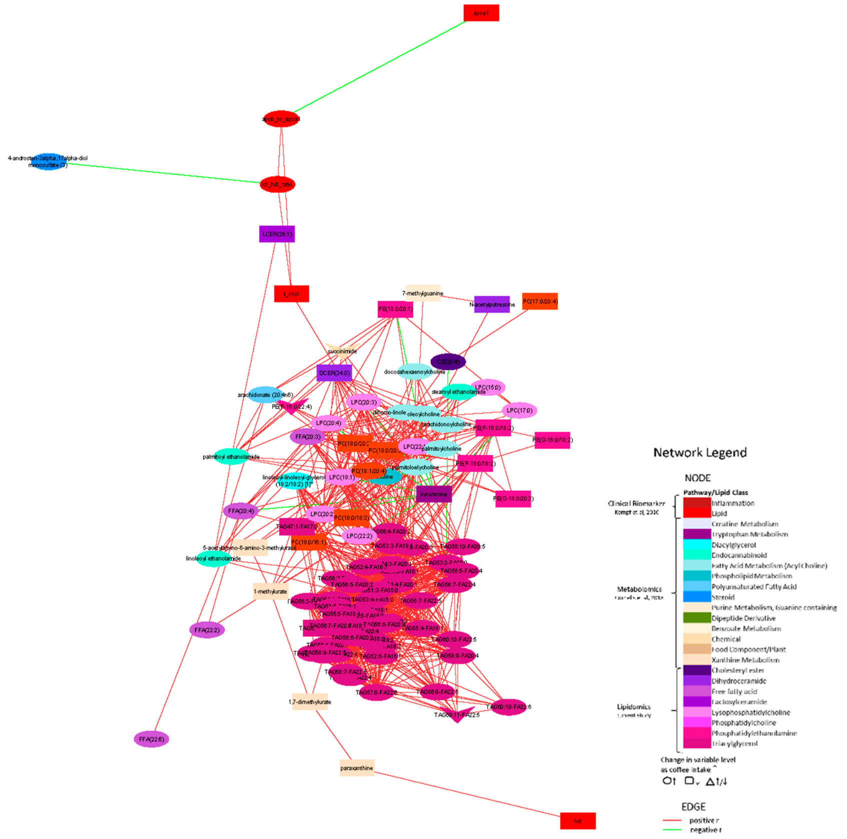
Network Legend
NODE
Pathway/Lipid Class
Clinical Biomarker
Inflammation
Lipid
Metabolomics
Creatine Metabolism
Tryptophan Metabolism
Diacylglycerol
Endocannabinoid
Fatty Acid Metabolism (Acyl Choline)
Phospholipid Metabolism
Polyunsaturated Fatty Acid
Steroid
Purine Metabolism, Guanine containing
Dipeptide Derivative
Benzoate Metabolism
Chemical
Food Component/Plant
Xanthine Metabolism
Lipidomics
Cholesteryl ester
Dihydroceramide
Free fatty acid
Lactosylceramide
Lysophosphatidylcholine
Phosphatidylcholine
Phosphatidylethanolamine
Triacylglycerol
Change in variable level as coffee intake
EDGE
positive r
negative r

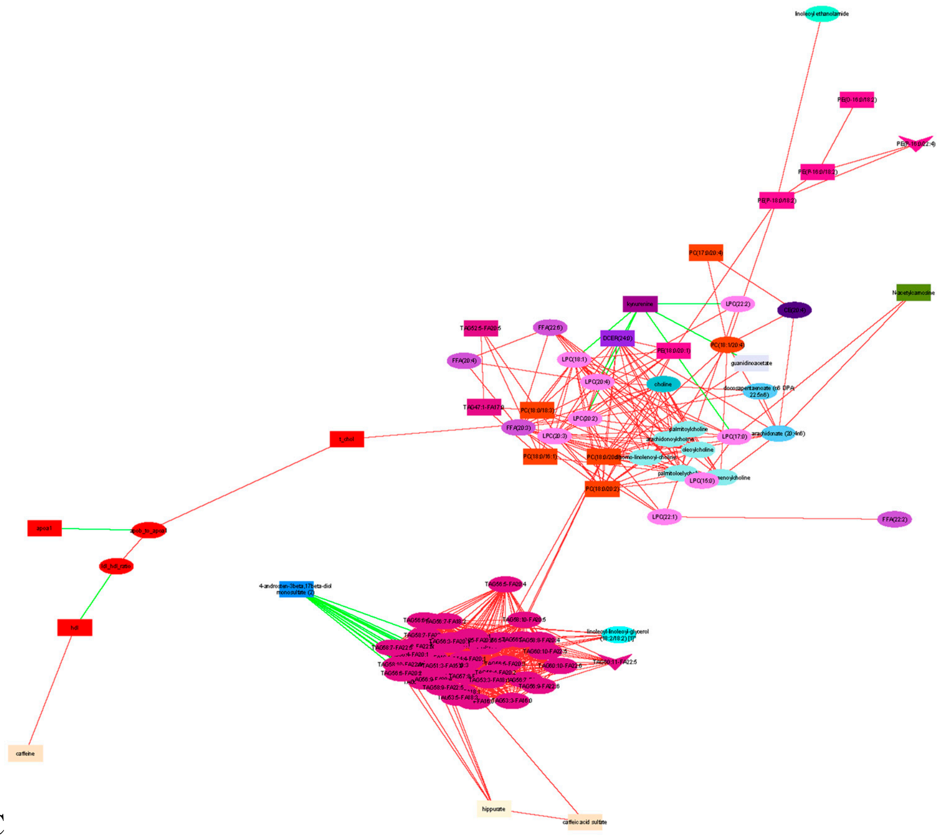

C

Figure S4.

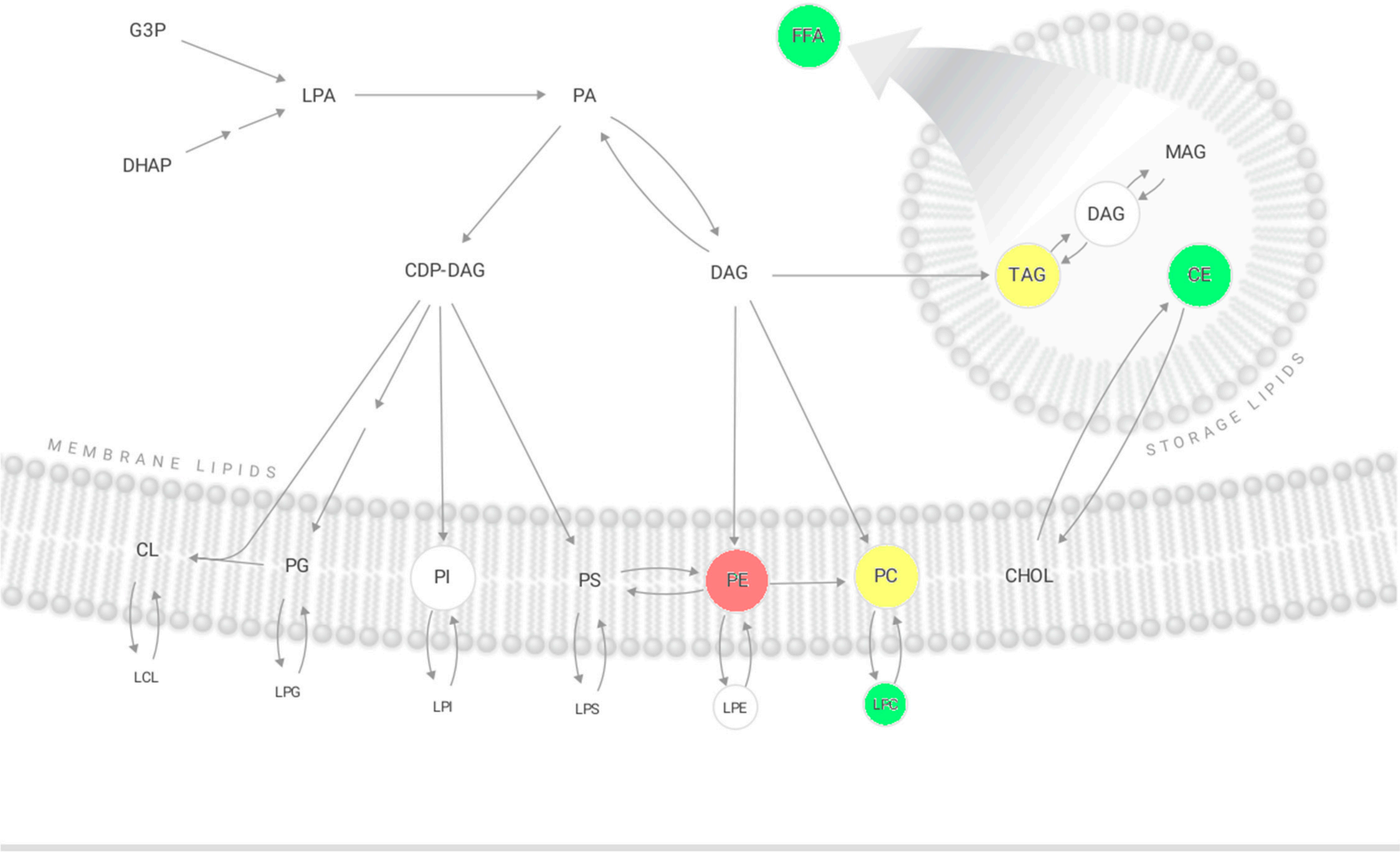

Lipid pathway including neutral and phospholipid lipid classes measured (circle nodes) in the current study. Lipid classes with lipid species that increased and decreased in response to coffee intake are colored red and green, respectively. Lipid classes with lipid species that increased or decreased are colored yellow. See Table 1 for details. CDP-DAG, CDP-diacylglycerol; CE, cholesteryl ester; CHOL, cholesterol; CL, cardiolipin; DAG, diacylglycerol; DHAP, dihydroxyacetone phosphate; FFA, free fatty acid; G3P, glycerol-3-phosphate; PA, phosphatic acid; PC, phosphatidylcholine; PE, phosphatidylethanolamine; PG, phosphatidylglycerol; PI, phosphatidylinositol; LCL, lysocardiolipin; LPA, lysophosphatidic acid; LPC, lysophosphatidylcholine; LPE, lysophosphatidylethanolamine; LPG, lysophosphatidylglycerol; LPI, lysophosphatidylinositol; LPS, lysophosphatidylserine; MAG, monoacylglycerol; TAG, triacylglycerol. Figure extracted from Metabolon's Surveyor software.

Figure S5.

A

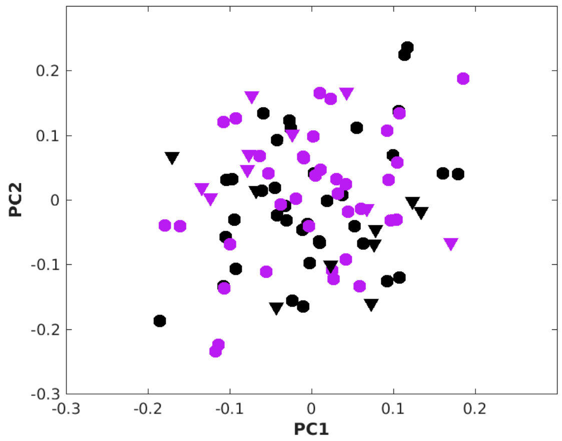

B

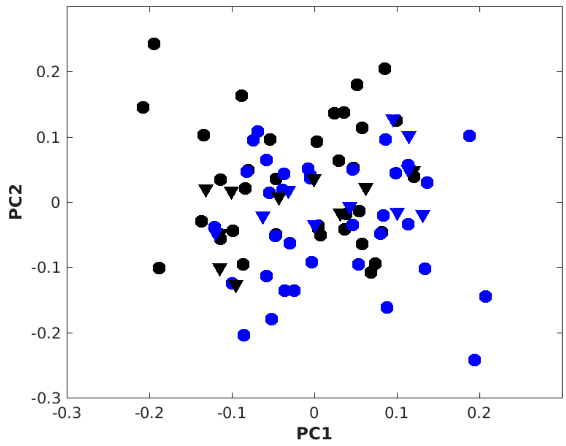

C

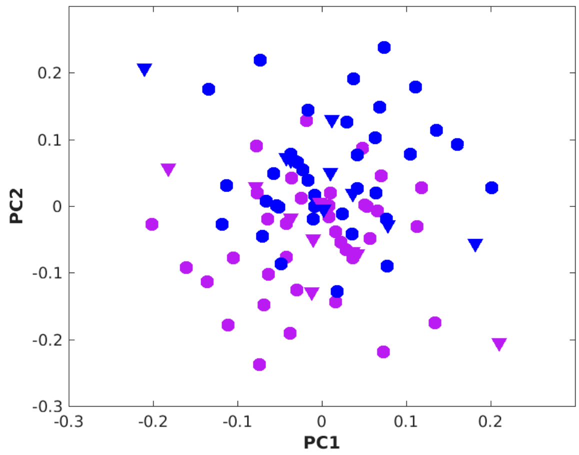

Multilevel PCA (A-C). Black, purple and blue PCA points represent samples measured after the 0 cups/d, 4 cups/d and 8 cups/d trial periods, respectively. Circle and triangle PCA shapes represent male and female

samples, respectively. MPLSDA score plots present overoptimistic representation of classification and thus are not presented as previously advocated [58].

Supplemental References

1. Kempf, K.; Herder, C.; Erlund, I.; Kolb, H.; Martin, S.; Carstensen, M.; Koenig, W.; Sundvall, J.; Bidel, S.; Kuha, S.*, et al.* Effects of coffee consumption on subclinical inflammation and other risk factors for type 2 diabetes: A clinical trial. *Am J Clin Nutr* **2010**, *91*, 950-957.
2. Westerhuis, J.A.; van Velzen, E.J.; Hoefsloot, H.C.; Smilde, A.K. Multivariate paired data analysis: Multilevel plsda versus oplsda. *Metabolomics* **2010**, *6*, 119-128.
3. Cornelis, M.C.; Erlund, I.; Michelotti, G.A.; Herder, C.; Westerhuis, J.A.; Tuomilehto, J. Metabolomic response to coffee consumption: Application to a three-stage clinical trial. *J Intern Med* **2018**.
4. Brereton, R.G.; Lloyd, G.R. Partial least squares discriminant analysis: Taking the magic away. *Journal of Chemometrics* **2014**, *28*, 213-225.
5. Altmaier, E.; Kastenmuller, G.; Romisch-Margl, W.; Thorand, B.; Weinberger, K.M.; Adamski, J.; Illig, T.; Doring, A.; Suhre, K. Variation in the human lipidome associated with coffee consumption as revealed by quantitative targeted metabolomics. *Mol Nutr Food Res* **2009**, *53*, 1357-1365.
6. Menni, C.; Zhai, G.; Macgregor, A.; Prehn, C.; Romisch-Margl, W.; Suhre, K.; Adamski, J.; Cassidy, A.; Illig, T.; Spector, T.D.*, et al.* Targeted metabolomics profiles are strongly correlated with nutritional patterns in women. *Metabolomics* **2013**, *9*, 506-514.
7. Floegel, A.; von Ruesten, A.; Drogan, D.; Schulze, M.B.; Prehn, C.; Adamski, J.; Pischon, T.; Boeing, H. Variation of serum metabolites related to habitual diet: A targeted metabolomic approach in epic-potsdam. *Eur J Clin Nutr* **2013**, *67*, 1100-1108.
8. Floegel, A.; Wientzek, A.; Bachlechner, U.; Jacobs, S.; Drogan, D.; Prehn, C.; Adamski, J.; Krumsiek, J.; Schulze, M.B.; Pischon, T.*, et al.* Linking diet, physical activity, cardiorespiratory fitness and obesity to serum metabolite networks: Findings from a population-based study. *Int J Obes (Lond)* **2014**.
9. Jacobs, S.; Kroger, J.; Floegel, A.; Boeing, H.; Drogan, D.; Pischon, T.; Fritsche, A.; Prehn, C.; Adamski, J.; Isermann, B.*, et al.* Evaluation of various biomarkers as potential mediators of the association between coffee consumption and incident type 2 diabetes in the epic-potsdam study. *Am J Clin Nutr* **2014**, *100*, 891-900.
10. Zheng, Y.; Yu, B.; Alexander, D.; Steffen, L.M.; Boerwinkle, E. Human metabolome associates with dietary intake habits among african americans in the atherosclerosis risk in communities study. *Am J Epidemiol* **2014**, *179*, 1424-1433.
11. Guertin, K.A.; Moore, S.C.; Sampson, J.N.; Huang, W.Y.; Xiao, Q.; Stolzenberg-Solomon, R.Z.; Sinha, R.; Cross, A.J. Metabolomics in nutritional epidemiology: Identifying metabolites associated with diet and quantifying their potential to uncover diet-disease relations in populations. *Am J Clin Nutr* **2014**, *100*, 208-217.
12. Playdon, M.C.; Sampson, J.N.; Cross, A.J.; Sinha, R.; Guertin, K.A.; Moy, K.A.; Rothman, N.; Irwin, M.L.; Mayne, S.T.; Stolzenberg-Solomon, R. Comparing metabolite profiles of habitual diet in serum and urine. *The American Journal of Clinical Nutrition* **2016**, ajcn135301.
13. Wang, Y.; Gapstur, S.M.; Carter, B.D.; Hartman, T.J.; Stevens, V.L.; Gaudet, M.M.; McCullough, M.L. Untargeted metabolomics identifies novel potential biomarkers of habitual food intake in a cross-sectional study of postmenopausal women. *J Nutr* **2018**.
14. Miranda, A.M.; Carioca, A.A.F.; Steluti, J.; da Silva, I.; Fisberg, R.M.; Marchioni, D.M. The effect of coffee intake on lysophosphatidylcholines: A targeted metabolomic approach. *Clinical nutrition (Edinburgh, Scotland)* **2017**, *36*, 1635-1641.
15. Barber, M.N.; Risis, S.; Yang, C.; Meikle, P.J.; Staples, M.; Febbraio, M.A.; Bruce, C.R. Plasma lysophosphatidylcholine levels are reduced in obesity and type 2 diabetes. *PLoS One* **2012**, *7*, e41456.
16. Heimerl, S.; Fischer, M.; Baessler, A.; Liebisch, G.; Sigruener, A.; Wallner, S.; Schmitz, G. Alterations of plasma lysophosphatidylcholine species in obesity and weight loss. *PloS one* **2014**, *9*, e111348.

17. Jee, S.H.; Kim, M.; Kim, M.; Yoo, H.J.; Kim, H.; Jung, K.J.; Hong, S.; Lee, J.H. Metabolomics profiles of hepatocellular carcinoma in a korean prospective cohort: The korean cancer prevention study-ii. *Cancer Prevention Research* **2018**, canprevres. 0249.2017.
18. Yin, X.; de Carvalho, L.P.; Chan, M.Y.; Li, S.F.Y. Integrated metabolomics and metallomics analyses in acute coronary syndrome patients. *Metallomics* **2017**, *9*, 734-743.
19. Zhong, H.; Fang, C.; Fan, Y.; Lu, Y.; Wen, B.; Ren, H.; Hou, G.; Yang, F.; Xie, H.; Jie, Z. Lipidomic profiling reveals distinct differences in plasma lipid composition in healthy, prediabetic and type 2 diabetic individuals. *GigaScience* **2017**.
20. Kühn, T.; Floegel, A.; Sookthai, D.; Johnson, T.; Rolle-Kampczyk, U.; Otto, W.; von Bergen, M.; Boeing, H.; Kaaks, R. Higher plasma levels of lysophosphatidylcholine 18: 0 are related to a lower risk of common cancers in a prospective metabolomics study. *BMC medicine* **2016**, *14*, 13.
21. Nestel, P.J.; Straznicky, N.; Mellett, N.A.; Wong, G.; De Souza, D.P.; Tull, D.L.; Barlow, C.K.; Grima, M.T.; Meikle, P.J. Specific plasma lipid classes and phospholipid fatty acids indicative of dairy food consumption associate with insulin sensitivity–. *The American journal of clinical nutrition* **2013**, *99*, 46-53.
22. Tonks, K.T.; Coster, A.C.; Christopher, M.J.; Chaudhuri, R.; Xu, A.; Gagnon-Bartsch, J.; Chisholm, D.J.; James, D.E.; Meikle, P.J.; Greenfield, J.R. Skeletal muscle and plasma lipidomic signatures of insulin resistance and overweight/obesity in humans. *Obesity* **2016**, *24*, 908-916.
23. Paapstel, K.; Kals, J.; Eha, J.; Tootsi, K.; Ottas, A.; Piir, A.; Jakobson, M.; Lieberg, J.; Zilmer, M. Inverse relations of serum phosphatidylcholines and lysophosphatidylcholines with vascular damage and heart rate in patients with atherosclerosis. *Nutrition, Metabolism and Cardiovascular Diseases* **2018**, *28*, 44-52.
24. Song, S.H.; Yoon, Y.; Park, K.U.; Song, J.; Kim, J.Q. Serum lysophosphatidylcholine level is not altered in coronary artery disease. *Clinical biochemistry* **2012**, *45*, 793-797.
25. Ressom, H.W.; Xiao, J.F.; Tuli, L.; Varghese, R.S.; Zhou, B.; Tsai, T.-H.; Ranjbar, M.R.N.; Zhao, Y.; Wang, J.; Di Poto, C. Utilization of metabolomics to identify serum biomarkers for hepatocellular carcinoma in patients with liver cirrhosis. *Analytica chimica acta* **2012**, *743*, 90-100.
26. Tulipani, S.; Palau-Rodriguez, M.; Alonso, A.M.; Cardona, F.; Marco-Ramell, A.; Zonja, B.; de Alda, M.L.; Muñoz-Garach, A.; Sanchez-Pla, A.; Tinahones, F.J. Biomarkers of morbid obesity and prediabetes by metabolomic profiling of human discordant phenotypes. *Clinica Chimica Acta* **2016**, *463*, 53-61.
27. Wang-Sattler, R.; Yu, Z.; Herder, C.; Messias, A.C.; Floegel, A.; He, Y.; Heim, K.; Campillos, M.; Holzapfel, C.; Thorand, B. Novel biomarkers for pre-diabetes identified by metabolomics. *Molecular systems biology* **2012**, *8*, 615.
28. Yang, S.J.; Kwak, S.-Y.; Jo, G.; Song, T.-J.; Shin, M.-J. Serum metabolite profile associated with incident type 2 diabetes in koreans: Findings from the korean genome and epidemiology study. *Scientific reports* **2018**, *8*, 8207.
29. Wittenbecher, C.; Mühlenbruch, K.; Kröger, J.; Jacobs, S.; Kuxhaus, O.; Floegel, A.; Fritsche, A.; Pischon, T.; Prehn, C.; Adamski, J. Amino acids, lipid metabolites, and ferritin as potential mediators linking red meat consumption to type 2 diabetes–. *The American journal of clinical nutrition* **2015**, *101*, 1241-1250.
30. Wahl, S.; Yu, Z.; Kleber, M.; Singmann, P.; Holzapfel, C.; He, Y.; Mittelstrass, K.; Polonikov, A.; Prehn, C.; Römisch-Margl, W. Childhood obesity is associated with changes in the serum metabolite profile. *Obesity facts* **2012**, *5*, 660-670.
31. Dorninger, F.; Moser, A.B.; Kou, J.; Wiesinger, C.; Forss-Petter, S.; Gleiss, A.; Hinterberger, M.; Jungwirth, S.; Fischer, P.; Berger, J. Alterations in the plasma levels of specific choline phospholipids in alzheimer's disease mimic accelerated aging. *Journal of Alzheimer's Disease* **2018**, *62*, 841-854.
32. Klavins, K.; Koal, T.; Dallmann, G.; Marksteiner, J.; Kemmler, G.; Humpel, C. The ratio of phosphatidylcholines to lysophosphatidylcholines in plasma differentiates healthy controls from patients with alzheimer's disease and mild cognitive impairment. *Alzheimer's & Dementia: Diagnosis, Assessment & Disease Monitoring* **2015**, *1*, 295-302.

33. Ganna, A.; Salihovic, S.; Sundstrom, J.; Broeckling, C.D.; Hedman, A.K.; Magnusson, P.K.; Pedersen, N.L.; Larsson, A.; Siegbahn, A.; Zilmer, M., *et al.* Large-scale metabolomic profiling identifies novel biomarkers for incident coronary heart disease. *PLoS Genet* **2014**, *10*, e1004801.
34. Guo, Y.; Wang, X.; Qiu, L.; Qin, X.; Liu, H.; Wang, Y.; Li, F.; Wang, X.; Chen, G.; Song, G. Probing gender-specific lipid metabolites and diagnostic biomarkers for lung cancer using fourier transform ion cyclotron resonance mass spectrometry. *Clinica chimica acta* **2012**, *414*, 135-141.
35. Pietilainen, K.H.; Sysi-Aho, M.; Rissanen, A.; Seppanen-Laakso, T.; Yki-Jarvinen, H.; Kaprio, J.; Oresic, M. Acquired obesity is associated with changes in the serum lipidomic profile independent of genetic effects--a monozygotic twin study. *PLoS One* **2007**, *2*, e218.
36. Reinehr, T.; Wolters, B.; Knop, C.; Lass, N.; Hellmuth, C.; Harder, U.; Peissner, W.; Wahl, S.; Grallert, H.; Adamski, J. Changes in the serum metabolite profile in obese children with weight loss. *European journal of nutrition* **2015**, *54*, 173-181.
37. Zhang, Y.; Liu, Y.; Li, L.; Wei, J.; Xiong, S.; Zhao, Z. High resolution mass spectrometry coupled with multivariate data analysis revealing plasma lipidomic alteration in ovarian cancer in asian women. *Talanta* **2016**, *150*, 88-96.
38. Wang, M.; Yang, R.; Dong, J.; Zhang, T.; Wang, S.; Zhou, W.; Li, H.; Zhao, H.; Zhang, L.; Wang, S. Simultaneous quantification of cardiovascular disease related metabolic risk factors using liquid chromatography tandem mass spectrometry in human serum. *Journal of Chromatography B* **2016**, *1009*, 144-151.
39. Rauschert, S.; Uhl, O.; Koletzko, B.; Kirchberg, F.; Mori, T.A.; Huang, R.-C.; Beilin, L.J.; Hellmuth, C.; Oddy, W.H. Lipidomics reveals associations of phospholipids with obesity and insulin resistance in young adults. *The Journal of Clinical Endocrinology & Metabolism* **2016**, *101*, 871-879.
40. Kim, J.Y.; Park, J.Y.; Kim, O.Y.; Ham, B.M.; Kim, H.J.; Kwon, D.Y.; Jang, Y.; Lee, J.H. Metabolic profiling of plasma in overweight/obese and lean men using ultra performance liquid chromatography and q-tof mass spectrometry (uplc-q-tof ms). *J Proteome Res* **2010**, *9*, 4368-4375.
41. Del Boccio, P.; Pieragostino, D.; Di Ioia, M.; Petrucci, F.; Lugaresi, A.; De Luca, G.; Gambi, D.; Onofrj, M.; Di Ilio, C.; Sacchetta, P. Lipidomic investigations for the characterization of circulating serum lipids in multiple sclerosis. *Journal of proteomics* **2011**, *74*, 2826-2836.
42. Dong, J.; Cai, X.; Zhao, L.; Xue, X.; Zou, L.; Zhang, X.; Liang, X. Lysophosphatidylcholine profiling of plasma: Discrimination of isomers and discovery of lung cancer biomarkers. *Metabolomics* **2010**, *6*, 478-488.
43. Jia, L.; Chen, J.; Yin, P.; Lu, X.; Xu, G. Serum metabonomics study of chronic renal failure by ultra performance liquid chromatography coupled with q-tof mass spectrometry. *Metabolomics* **2008**, *4*, 183-189.
44. Okita, M.; Gaudette, D.C.; Mills, G.B.; Holub, B.J. Elevated levels and altered fatty acid composition of plasma lysophosphatidlycholine (lysopc) in ovarian cancer patients. *International journal of cancer* **1997**, *71*, 31-34.
45. Sutter, I.; Klingenberg, R.; Othman, A.; Rohrer, L.; Landmesser, U.; Heg, D.; Rodondi, N.; Mach, F.; Windecker, S.; Matter, C.M. Decreased phosphatidylcholine plasmalogens–a putative novel lipid signature in patients with stable coronary artery disease and acute myocardial infarction. *Atherosclerosis* **2016**, *246*, 130-140.
46. Zhao, Z.; Xiao, Y.; Elson, P.; Tan, H.; Plummer, S.J.; Berk, M.; Aung, P.P.; Lavery, I.C.; Achkar, J.P.; Li, L. Plasma lysophosphatidylcholine levels: Potential biomarkers for colorectal cancer. *Journal of clinical oncology* **2007**, *25*, 2696-2701.
47. Zhao, X.; Fritsche, J.; Wang, J.; Chen, J.; Rittig, K.; Schmitt-Kopplin, P.; Fritsche, A.; Haring, H.U.; Schleicher, E.D.; Xu, G., *et al.* Metabonomic fingerprints of fasting plasma and spot urine reveal human pre-diabetic metabolic traits. *Metabolomics* **2010**, *6*, 362-374.
48. Graessler, J.; Schwudke, D.; Schwarz, P.E.; Herzog, R.; Shevchenko, A.; Bornstein, S.R. Top-down lipidomics reveals ether lipid deficiency in blood plasma of hypertensive patients. *PloS one* **2009**, *4*, e6261.

49. Sigruener, A.; Kleber, M.E.; Heimerl, S.; Liebisch, G.; Schmitz, G.; Maerz, W. Glycerophospholipid and sphingolipid species and mortality: The ludwigshafen risk and cardiovascular health (luric) study. *PloS one* **2014**, *9*, e85724.
50. Sutphen, R.; Xu, Y.; Wilbanks, G.D.; Fiorica, J.; Grendys, E.C.; LaPolla, J.P.; Arango, H.; Hoffman, M.S.; Martino, M.; Wakeley, K. Lysophospholipids are potential biomarkers of ovarian cancer. *Cancer Epidemiology and Prevention Biomarkers* **2004**, *13*, 1185-1191.
51. Cheng, M.-L.; Chang, K.-H.; Wu, Y.-R.; Chen, C.-M. Metabolic disturbances in plasma as biomarkers for huntington's disease. *The Journal of nutritional biochemistry* **2016**, *31*, 38-44.
52. Fernandez, C.; Sandin, M.; Sampaio, J.L.; Almgren, P.; Narkiewicz, K.; Hoffmann, M.; Hedner, T.; Wahlstrand, B.; Simons, K.; Shevchenko, A. Plasma lipid composition and risk of developing cardiovascular disease. *PloS one* **2013**, *8*, e71846.
53. Liu, X.; Li, J.; Zheng, P.; Zhao, X.; Zhou, C.; Hu, C.; Hou, X.; Wang, H.; Xie, P.; Xu, G. Plasma lipidomics reveals potential lipid markers of major depressive disorder. *Analytical and bioanalytical chemistry* **2016**, *408*, 6497-6507.
54. Graessler, J.; Bornstein, T.; Goel, D.; Bhalla, V.; Lohmann, T.; Wolf, T.; Koch, M.; Qin, Y.; Licinio, J.; Wong, M.-L. Lipidomic profiling before and after roux-en-y gastric bypass in obese patients with diabetes. *The pharmacogenomics journal* **2014**, *14*, 201.
55. Krautbauer, S.; Eisinger, K.; Wiest, R.; Liebisch, G.; Buechler, C. Systemic saturated lysophosphatidylcholine is associated with hepatic function in patients with liver cirrhosis. *Prostaglandins & other lipid mediators* **2016**, *124*, 27-33.
56. Fonteh, A.N.; Chiang, J.; Cipolla, M.; Hale, J.; Diallo, F.; Chirino, A.; Arakaki, X.; Harrington, M.G. Alterations in cerebrospinal fluid glycerophospholipids and phospholipase a2 activity in alzheimer's disease. *Journal of lipid research* **2013**, jlr. M037622.
57. Poole, R.; Kennedy, O.J.; Roderick, P.; Fallowfield, J.A.; Hayes, P.C.; Parkes, J. Coffee consumption and health: Umbrella review of meta-analyses of multiple health outcomes. *bmj* **2017**, *359*, j5024.
58. Westerhuis, J.A.; Hoefsloot, H.C.; Smit, S.; Vis, D.J.; Smilde, A.K.; van Velzen, E.J.; van Duijnhoven, J.P.; van Dorsten, F.A. Assessment of plsda cross validation. *Metabolomics* **2008**, *4*, 81-89.
